# Hfq protein and GcvB small RNA tailoring of *oppA* target mRNA to levels allowing translation activation by MicF small RNA in *Escherichia coli*

**DOI:** 10.1080/15476286.2023.2179582

**Published:** 2023-03-01

**Authors:** Marie-Claude Carrier, David Lalaouna, Eric Massé

**Affiliations:** Department of Biochemistry and Functional Genomics, RNA Group, Université de Sherbrooke, Sherbrooke, Québec, Canada

**Keywords:** sRNA interactome, ABC transporter, translation enhancer, RNA affinity purification, mechanism of action, sRNA chaperone

## Abstract

Traffic of molecules across the bacterial membrane mainly relies on porins and transporters, whose expression must adapt to environmental conditions. To ensure bacterial fitness, synthesis and assembly of functional porins and transporters are regulated through a plethora of mechanisms. Among them, small regulatory RNAs (sRNAs) are known to be powerful post-transcriptional regulators. In *Escherichia coli*, the MicF sRNA is known to regulate only four targets, a very narrow targetome for a sRNA responding to various stresses, such as membrane stress, osmotic shock, or thermal shock. Using an *in vivo* pull-down assay combined with high-throughput RNA sequencing, we sought to identify new targets of MicF to better understand its role in the maintenance of cellular homoeostasis. Here, we report the first positively regulated target of MicF, the *oppA* mRNA. The OppA protein is the periplasmic component of the Opp ATP-binding cassette (ABC) oligopeptide transporter and regulates the import of short peptides, some of them bactericides. Mechanistic studies suggest that *oppA* translation is activated by MicF through a mechanism of action involving facilitated access to a translation-enhancing region in *oppA* 5ʹUTR. Intriguingly, MicF activation of *oppA* translation depends on cross-regulation by negative trans-acting effectors, the GcvB sRNA and the RNA chaperone protein Hfq.

## Introduction

Bacterial cells are in direct contact with their environment, which can be harsh and unpredictable. To protect themselves against the extracellular medium, bacteria have developed a complex and layered structure, the cell envelope. In Gram-negative bacteria, this envelope is composed of three main elements: the inner membrane (IM), the peptidoglycan-containing periplasm and the outer membrane (OM) [1].

The periplasm is a dense, viscous and oxidizing cellular compartment containing more than 60 known proteins [2]. Previously, several groups have focused on the quality-control role of the periplasm, identifying folding chaperones such as SurA [3] and Skp [4], and unravelling the chaperone properties of the protease DegP [5]. Since then, many other proteins, such as the substrate-binding protein OppA, have been shown to also possess chaperone activity [6,7].

Due to their crucial importance for cellular membrane integrity, viability, signal transduction and virulence, the expression of membrane proteins and transporters is tightly regulated [8]. Membrane proteins are often under the control of transcriptional regulators responding to specific stresses [9]. Post-translational control of membrane proteins occurs xmainly in the periplasm, where misfolded proteins can be degraded by proteases such as DegP [10–12]. Both membrane proteins and transporters can also be regulated at the post-transcriptional level by small regulatory RNAs (sRNAs) [8,13,14].

sRNAs are usually non-coding RNAs, which can either originate from their own promoter in intergenic regions or derive from 5’ [15,16] and 3ʹUTRs [17,18], the coding sequence (CDS) of mRNA transcripts, or other non-coding RNAs such as tRNAs [19]. sRNAs can negatively or positively regulate their target mRNA stability and/or translation [20–22]. Negative regulation mostly occurs by hindering translation initiation and favouring mRNA degradation. In contrast, positive regulation generally results from the disruption of inhibitory secondary structures in the mRNA 5′UTR, facilitating translation initiation. sRNAs often act in concert with a chaperone protein, such as Hfq [23–27]. A plethora of new and unconventional regulatory mechanisms have been emerging over the last few years. For example, sRNAs are capable of regulation through base-pairing in the coding region of their targets, or they can even target translation enhancer elements to modulate protein synthesis.

Many sRNAs are well-known membrane-stress response effectors. In *E. coli*, RybB, MicF, MicA, MicC and RseX are responsible for the coordinated repression of porin-encoding mRNAs *ompF, ompC, ompW* and *ompA* during membrane stress [28]. Additional sRNAs, such as GcvB, also repress membrane proteins and transporters [29]. When glycine is abundant, GcvB expression is induced through the GcvA regulator and represses more than 30 target mRNAs, including *dppA, tcyJ* and *oppA*, all encoding subunits of ABC transporters [30,31].

The MicF sRNA was identified decades ago by the group of Inouye as a repressor of *ompF* expression [32]. Since then, its targetome in *E. coli* has been expanded by only three repressed mRNAs: two transcriptional regulators, *lrp* and *cpxR* and *phoE*, which encodes a porin importing phosphorus-containing compounds [33].

Here, we combined results from multiple-screening assays to identify new target mRNAs of MicF to better understand its role during membrane stress [34]. Data from both high-throughput techniques MAPS (MS2 affinity purification coupled with RNA sequencing) and RIL-Seq (RNA interaction by ligation and sequencing) [35] were combined together with *in silico* predictions from CopraRNA [36–38] to identify two new targets of MicF in *E. coli*, namely *tcyJ* (formerly *fliY*) and *oppA* mRNAs. Notably, both newly identified targets of MicF are also targeted by the GcvB sRNA. Our results indicate that MicF increases *oppA* mRNA translational activity only in the presence of both translational repressors GcvB and Hfq. Additional *in vivo* and *in vitro* data indicate that MicF promotes translation initiation of *oppA* mRNA probably by rendering accessible a translational enhancer site in the 5ʹUTR. Translation activation of *oppA* mRNA depends on the presence of active negative regulators and suggests an unusual interplay between opposite effectors.

## Material and methods

### Reagents

Enzymes EcoRI (R3101), BamHI (R3136), SphI (R3182), MscI (R0534), T4 Polynucleotide kinase (M0201), Protoscript II (M0368), calf intestinal phosphatase (M0290), Q5 High fidelity DNA polymerase (M0491), XRN-1 (M0338) and Vent (exo–) DNA Polymerase (M0257) were purchased from New England Biolabs (Ipswich, MA, USA). Ampicillin (AMP201.5), Chloramphenicol (CLR201.5), kanamycin (KAN201.5), Ortho-Nitrophenyl-β-galactoside (ONP301.5), Polymyxin B (POL435.5), tetracyclin (TET701) and Phenol (PHE509.1) were purchased from BioShop (Burlington, ON, Canada). Acrylamide:Bisacrylamide 19:1 and 29:1 (A0006 and A0007) and PCR product purification kit (BS-365) were purchased from BioBasic (Markham, ON, Canada). Turbo DNase (AM2238) was purchased from ThermoFisher Scientific (Waltham, MA, USA). QIAprep Spin Miniprep Kit was purchased from QIAGEN (Hilden, Germany). Pyrophosphatase (10108987001) was purchased from Millipore Sigma (Burlington, MA, USA). T4 DNA ligase and RNase Inhibitor were produced by the Sherbrooke University Protein Purification Platform. T7 RNA polymerase was purified in house (Massé Laboratory).

### Biological resources

All strains are described in Supplementary Table S1. All plasmids and oligonucleotides (oligos) are described in Supplementary Table S2 and Supplementary Table S3, respectively. For construction of *lacZ* fusions, PCR fragments were obtained with oligos EM4513-EM1321 (*oppA*), EM1320-EM1321 (P2_*oppA*), EM4513-EM5064 (P1_*oppA*_prom_), EM3895-EM3896 (*ompF*) or EM4860-EM4861 (*yjbJ*). The PCR fragments were digested with EcoRI and BamHI and ligated into an EcoRI/BamHI-digested pRS1551 (translational fusion) or an EcoRI/BamHI-digested pFRΔ (transcriptional fusion). To obtain *oppA*_UGC_, a three-step PCR was performed with oligos EM4513-4467 (PCR1), EM4466-EM1321 (PCR2) and EM4513-EM1321 (PCR3) using PCR1+ PCR2 as a template. PCR3 was digested with EcoRI and BamHI and ligated into an equally digested pRS1551. To obtain *oppA*_Δ399_, a three-step PCR was performed with oligos EM4513-5096 (PCR4), EM5095-EM1321 (PCR5) and EM4513-EM1321 (PCR6) using PCR4+ PCR5 as a template. PCR6 was digested with EcoRI and BamHI and ligated into an equally digested pRS1551. All fusions were then inserted into the chromosome of relevant strains at the λ *attI* site [39]. Lysogens were screened by PCR for selection of single insertion recombinant using oligos EM111‐EM112‐EM113 and then sequenced using oligos EM194-195 [40].

Plasmid pBAD-*micF*_CGA_ was generated by digestion of a PCR fragment (oligos EM4426-EM2681) with MscI and SphI followed by ligation into an equally digested pNM12 vector. Plasmids pFRΔ-*oppA*+term and pFRΔ-*oppA*-MS2+ term were obtained by first performing sequential PCR reactions with oligos EM4513-EM3996 (PCR7, *oppA*+term), EM4513 + 1577 (PCR8 on PCR7, *oppA*+term), EM4513-EM3995 (PCR9, *oppA*-MS2+ term), EM4513-EM1576 (PCR10 on PCR9, *oppA*-MS2+ term) and EM4513-EM1577 (PCR11 on PCR10, *oppA*-MS2+ term). PCR8 and PCR11 were then digested with EcoRI and BamHI and ligated into an equally digested pFRΔ vector.

Chromosomal mutations or knockouts were obtained as previously described [41]. For *gcvB*ΔR1::*cat*, PCR12 was first performed on pBAD-*gcvB*ΔR1 with oligos EM168-EM4587. Then, PCR13 was performed on PCR12 with oligos EM4592-EM4587. Finally, PCR13 was used as a primer, along with oligo EM4588 on pKD3 to generate PCR14. For generation of Δ*lrp::cat*, PCR15 was performed on pKD3 with oligos EM3647-EM3648. For generation of *oppA*-1xFLAG::*kan*, PCR16 was performed with oligos EM4393-EM4091 on pKD4. Then, PCR17 was performed with oligos EM4394-EM4091 using PCR16 as a template. PCR14, PCR15 and PCR17 were transformed into a wild-type (WT) strain expressing the λ Red system from the pKD46 plasmid [41].

Chromosomal deletion of *gcvB* (*gcvB::tet*) was obtained as follows. First, PCR18 was performed on strain GD641 with oligos EM215-EM216 to amplify the tetracycline resistance cassette. Then, PCR19 was performed with oligos EM1247-EM1248 using PCR18 as a template. PCR19 was transformed into DY330 after induction of λ‐Red [42].

The Hfq-link-*kan* constructs were obtained as follows. PCR20 was performed on pKD4 with oligos EM4835-4836 and transformed into a WT strain, expressing the λ Red system from the pKD46 plasmid [41] to give rise to strain KP2242. The Hfq-link-*kan* construct was transferred to a WT strain (EM1055) by P1 transduction (KP2258). For the Hfq Y25D-link-*kan* construct, PCR21 was performed on strain KP2242 with oligos EM1810-EM1690. PCR22 was then carried out on PCR21 with oligos EM1810-4473. PCR22 was transformed into a Hfq Y25D strain expressing the λ Red system from the pKD46 plasmid [41] to give rise to strain KP2257. The Hfq Y25D-link-*kan* construct was transferred to a WT strain (EM1055) by P1 transduction (KP2261).

The addition of the Flag tag to Hfq was done by first performing PCR23 on pKD4 using oligos EM1689-EM1690. PCR24 was then performed on PCR23 using oligos EM1689-EM1691. PCR24 was transformed into DY330 after induction of λ‐Red [42].

For Hfq Y25D-3x-Flag construct, PCR25 was carried on pKD4 with oligos EM1689-EM1690. PCR26 was performed on PCR23 with oligos EM1691-EM1690. PCR26 was transformed into the Hfq Y25D strain expressing the λ Red system from the pKD46 plasmid [41]. The Hfq Y25D-3x-Flag construct was transferred to a WT strain (EM1055) by P1 transduction (KP1758).

Strains were selected with chloramphenicol, kanamycin, or tetracycline and verified by PCR. Transfer of all chromosomal mutations to different strains was achieved by P1 transduction, and recombinants were selected with appropriate antibiotics and verified by PCR. When necessary, FRT-flanked antibiotic resistance cassettes were eliminated after transformation with pCP20 (FLIP), as described before [41].

### Growth conditions

All strains derive from *Escherichia coli* strain K-12 MG1655. Unless stated otherwise, the cells were grown in Luria-Bertani (LB) medium at 37°C. When required, the medium was supplemented with 50 μg/ml ampicillin, 30 μg/ml chloramphenicol, or 10 μg/ml tetracycline.

### MS2 affinity purification couples with RNA sequencing (MAPS)

The MAPS was performed as described before [19,43,44]. The *rne131* background was used to maximize target recovery. Cells grown in the LB medium were harvested at OD_600nm_ = 0.5 and 1.0 following induction of either pBAD-*micF* or pBAD-MS2-*micF* expression with 0.1% arabinose for 15 min. Raw data was analysed as described previously, with the only modification being that due to the low quality of reads in the R2 sequencing dataset, analysis was done single-ended using R1 [43,44]. Selection of the putative targets was done according to the following cut-offs: RNAs with >25 reads (before normalization) and a ratio ≥3 were selected. Among these, only the RNAs with >50 reads (before normalization) and a ratio of <6.5 in the pBAD-MS2 control were selected (Supplementary Data Table S4). GalaxyProject [45] and UCSC Microbial Genome Browser [46] were used to analyse and visualize the data. MS2*‐micF* GEO accession number is GSE113584. MS2-control was previously published with GEO accession number GSE67606 [47]. For MS2 affinity purification, followed by Northern blot analysis in different backgrounds (WT, Δ*gcvB, hfq* Y25D), cells were harvested at OD_600nm_ = 2.0. Input samples were taken prior to any manipulation of the culture. The output samples result from MS2 affinity purification. RNA was analysed by Northern blot as described below.

### β-Galactosidase assays

β-Galactosidase assays were performed as described previously [48]. Cells from overnight cultures were diluted 1/1000 in 50 ml fresh medium and grown at 37°C with agitation. Antibiotics were used at appropriate concentrations when required. MicF was expressed either endogenously or from a BAD promoter induced by addition of 0.25–0.75% arabinose at OD_600nm_ = 0.5, as specified. Results are presented as mean ± Standard Deviation (mean ± SD) of the V_max_/OD_600nm_ calculated values.

### Total RNA extraction and Northern blot analysis

Cells were grown in the LB medium at 37°C, and sample were collected at different OD_600nm_. For *rne3071* experiments, cultures were grown at 30°C until an OD_600nm_ = 2.0 before half was transferred at 44°C for 15 minutes before sampling. For half-life assays, cells were treated with 250 µg/ml Rifampicine before total RNA extraction was performed at specific time points post-induction. Total RNA was extracted following the hot phenol procedure described previously [49]. For polyacrylamide gels, 5 μg RNA was loaded on 5–10% acrylamide 29:1, 8 M urea gels and migrated at 100 V. RNAs were electro-transferred to Hybond‐XL membranes. Membranes were prehybridized in Church buffer (0.36 M Na_2_HPO_4_, 0.14 M NaH_2_PO_4_, 1 mM EDTA, 7% SDS) [50] at 42°C and radiolabeled DNA probes were then added. After incubation, membranes were washed, exposed to phosphor screens and visualized using Typhoon Trio (GE Healthcare) instrument. Image Studio Lite software (LICOR) was used for densitometry analysis when applicable.

### In vitro transcription

Templates for *in vitro* transcriptions were generated by PCR using oligos EM4528-EM3438 (T7-*oppA*), EM2982-EM2983 (T7-*micF*), EM1215-EM1216 (T7-*gcvB*), EM4436-EM2983 (T7-*micF_CGA_*) and ΕΜ4528-ΕΜ4665 (T7-*oppA*_DRBS_) on genomic DNA. T7-*oppA*_UCG_ was obtained by performing a three-step mutagenesis. PCR1 (EM4528-EM4467) and PCR2 (EM4466-EM3438) were performed on genomic DNA and served as a template for PCR3 (EM4528-EM3438). T7-*oppA*_Δ399_ was obtained by performing a three-step mutagenesis. PCR4 (EM4528-EM5096) and PCR5 (EM5095-EM3438) were performed on genomic DNA and served as a template for PCR6 (EM4528-EM3438). Transcription reactions were carried out as described previously [51]. When necessary, RNA fragments were dephosphorylated with calf intestinal phosphatase and 5’-radiolabeled with γ-^32^P using T4 polynucleotide kinase (NEB).

### Lead acetate probing assays

Lead acetate probing assay was performed as described previously, with modifications [51]. About 0.2 pmol of 5’-radiolabeled *oppA* RNA was incubated with or without 1 μM MicF sRNA for 10 min at 37°C before treatment with 10 mM PbAc for 2 min. H_2_O was used for controls. Reactions were stopped by the addition of 10 μl Loading Buffer II (LBII: 95% formamide, 18 mM EDTA, 0.025% SDS, 0.025% xylene cyanol-bromophenol blue). For in-line probing, 0.2 pmol 5’-radiolabeled *oppA* RNA was incubated with or without 1 μM MicF sRNA for 46 h at 22°C in in-line probing buffer (50 mM Tris-Cl pH 8.0, 100 mM KCl, 25 mM MgCl_2_). Reactions were stopped by the addition of 10 μl LBII. Samples were migrated on polyacrylamide gels (8% acrylamide:bisacrylamide 19:1, 8 M urea) in TBE 1X at 38 W. Gels were dried, exposed to phosphor screens and visualized using the Typhoon Trio (GE Healthcare) instrument.

### Footprint assays

Footprinting assays were performed as described previously, with modifications [51]. About 0.2 pmol of 5’-radiolabeled *oppA* RNA was incubated with or without 1 μM purified Hfq for 10 min at 37°C before treatment with 10 mM PbAc for 2 min. H_2_O was used for controls. Reactions were stopped by addition of STOP solution (50 mM Tris-Cl pH 8.0, 0.1% SDS). RNA was extracted using phenol-chloroform. Samples were migrated on polyacrylamide gels (8% acrylamide:bisacrylamide 19:1, 8 M urea) in TBE 1X at 38 W. Gels were dried, exposed to phosphor screens and visualized using the Typhoon Trio (GE Healthcare) instrument.

### Electrophoretic mobility shift assays

Electrophoretic mobility shift assays (EMSAs) were performed as described by Morita and colleagues, with slight modifications [52]. Radiolabeled *oppA* or *oppA*_UCG_ RNA was heated for 1 min at 90°C and put on ice for 1 min. The RNA was diluted at 5 nM or 20 nM, as indicated, in binding buffer (10 nM Tris-HCl pH 8.0, 1 mM DTT, 1 mM MgCl_2_, 20 mM KCl, 10 mM Na_2_HPO_4_-NaH_2_PO_4_ pH 8.0, 12.5 μg/mL yeast tRNA) and mixed with specific concentrations of MicF (0–500 nM). Samples were incubated for 15 min at 37°C, and reactions were stopped by addition of 1 μL of non-denaturing loading buffer (1X TBE, 50% glycerol, 0.1% bromophenol blue, 0.1% xylene cyanol). Samples were resolved on native polyacrylamide gels (5% acrylamide:bisacrylamide 29:1) in cold TBE 1X and migrated at 50 V, at 4°C. Gels were dried, exposed to phosphor screens and visualized using the Typhoon Trio (GE Healthcare) instrument. Image Studio Lite software (LICOR) was used for densitometry analysis when required.

### Primer extension assays

Reverse transcription (RT) was carried out with radiolabeled primer EM4516 or EM4518 on 20 μg of total RNA extracted from the *rne3071* mutant strain at 30°C and at 44°C or from the WT and Δ*micF* strains at 37°C following a previously described protocol [48]. Briefly, RNA was incubated 5 min at 65°C in the presence of the primer and dNTPs. Reaction was cooled down, and an RNase inhibitor (in house) and ProtoScript II (NEB) were added to the reactions. RT was carried out for 1 h at 42°C before inactivation of the enzyme 10 min at 90°C. The resulting radioactive complementary DNA was migrated on polyacrylamide gels (8% acrylamide:bisacrylamide 19:1, 8 M urea) in TBE 1X at 38 W. Gels were dried, exposed to phosphor screens and visualized using the Typhoon Trio (GE Healthcare) instrument.

### Toeprinting assays

Toeprinting assays were performed as described previously [53]. RNAs were transcribed from PCR products described above. Radiolabeled primer EM3551 and ProtoScript II (NEB) were used for reverse transcription. The resulting radioactive complementary DNA was migrated on polyacrylamide gels (8% acrylamide:bisacrylamide 19:1, 8 M urea) in TBE 1X at 38 W. Gels were dried, exposed to phosphor screens and visualized using the Typhoon Trio (GE Healthcare) instrument.

### Computational resources

The following online resources have been used in this study:

CopraRNA (http://rna.informatik.uni-freiburg.de/CopraRNA/Input.jsp) [36–38], UCSC Microbial Genome Browser (http://microbes.ucsc.edu/) [46], GalaxyProject (https://usegalaxy.org/) [45], GWIPS-viz (http://gwips.ucc.ie/) [54].

### Statistical analyses

The number of biological replicates (N) is indicated for each experiment in figure caption. When applicable, statistical analyses were performed using an unpaired two-tailed Student’s *t* test or a two-way ANOVA multiple-comparison test (adjusted *P* value) on the GraphPad Prism 9 software, as detailed in each figure caption. For all experiments, *P* < 0.05 was considered statistically significant. Specific *P* values are indicated in the figure caption of each experiment, when applicable.

## Results

### In vivo analysis of MicF targetome reveals new mRNA targets

**Figure 1.**
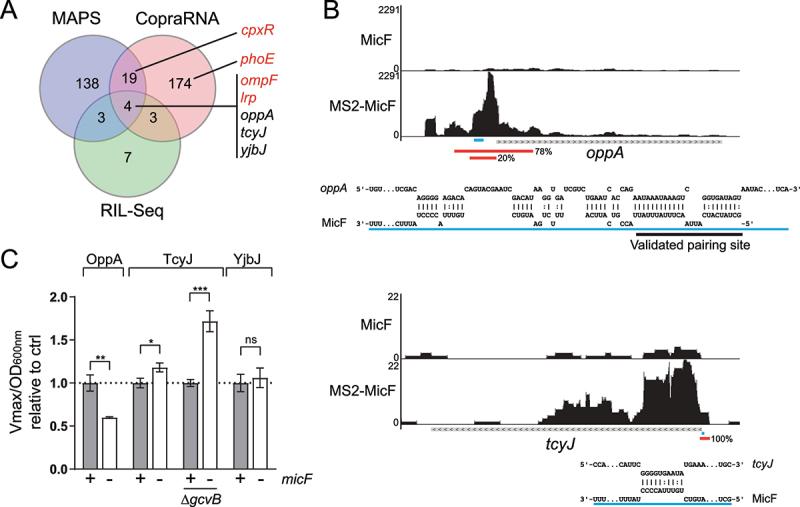
Identification of new targets of MicF. (A) Overlap of putative MicF targets obtained from RIL-Seq, CopraRNA and MAPS (enrichment ≥ 3, reads ≥ 25) experiments represented in a Venn Diagram. Previously characterized targets of MicF are indicated in red. **(B)** Visualization of MicF and MS2-MicF MAPS reads of *oppA* (top) and *tcyJ* (bottom) using the UCSC microbial genome browser (http://archaea.ucsc.edu) [46]. CopraRNA-predicted base-pairing sites are represented below each graphical representation of RNA-seq reads. Blue lines represent predicted base-pairing sites. Red lines represent fragments recovered by RIL-Seq [35]. For *oppA*, the validated pairing site (see Fig. 2A) is underlined in black. **(C)** Translational β-galactosidase assay of OppA-LacZ in WT and Δ*micF*, of TcyJ-LacZ in WT, Δ*micF*, Δ*gcvB* and Δ*micF*Δ*gcvB* and of YjbJ-LacZ in WT and Δ*micF*. Samples (N = 3, mean ± SD) were taken at OD_600nm_ = 2.5. *p = 0.01, **p = 0.0019, ***p = 0.0007, ns: *p* > 0.05, unpaired two-tailed Student’s *t* test.

We previously used the MAPS technique to identify new target mRNAs of various sRNAs [19,30,47,55]. Here, we tagged the 5’ end of the MicF sRNA with an MS2 RNA aptamer, expressed the construct in a Δ*micF* strain and proceeded to affinity purify the MS2-tagged MicF sRNA. Experiments were performed in a *rne131* background, a mutation truncating the C-terminal of RNase E that serves as the scaffold for assembly of the RNA degradosome complex [56]. The *rne131* background was used to limit the degradation of sRNA-mRNA complexes. Samples taken during the exponential growth phase and the early stationary growth phase were combined for the purification steps. After affinity purification, RNA sequencing was performed. Enriched RNAs were identified as described previously [43,44]. Data analysis revealed that three of the four previously characterized targets of MicF were enriched (*cpxR, ompF* and *lrp*), as well as new putative targets (Supplementary Table S4). The fourth previously characterized target of MicF, *phoE*, might not be expressed in our experimental conditions. Considering the high number of hits obtained, we compared the MAPS data to *in silico* interaction predictions obtained with the CopraRNA algorithm [34,36–38]. Also, we compared both data sets (MAPS and CopraRNA) to the RIL-Seq data obtained from the stationary phase condition [34,35]. RIL-Seq exploits the role of Hfq in sRNA-dependent regulation to identify sRNA-mRNA complexes. We observed a slight overlap of putative targets between the different methods, with only five mRNAs identified by all three: *ompF, lrp, oppA, tcyJ* and *yjbJ* (Fig. 1A, Supplementary Table S4). Two of those RNAs (*ompF* and *lrp*) are previously characterized targets of MicF, suggesting that combining these methods can efficiently identify sRNA targets [34]. We therefore focused on *oppA, tcyJ* and *yjbJ* mRNAs as new candidate targets of MicF.

OppA is the main periplasmic substrate-binding protein (SBP) of the Opp ABC transporter, responsible for the import of oligopeptides. Similarly, *tcyJ* encodes the periplasmic SBP of the cystine ABC transporter [57]. The location of MAPS reads enrichment on both *oppA* and *tcyJ* mRNAs correlate with CopraRNA base-pairing predictions, suggesting that MicF pairs with *oppA* mRNA at more than 100 nucleotides upstream of the initiator codon, and *tcyJ* at the RBS sequence (Fig. 1B). Notably, both *oppA* and *tcyJ* mRNAs are targeted by the sRNA GcvB (Fig. S1A, B) [29,30]. The reads profile for the *yjbJ* mRNA did not allow base-pairing prediction, but CopraRNA locates a putative base-pairing site of MicF in *yjbJ* CDS, outside of the five-codon window [58]. The role of YjbJ has not been characterized yet.

We constructed translational fusions of *oppA, tcyJ* and *yjbJ* genes to the *lacZ* reporter and performed β-galactosidase assays in both wild-type (WT) and Δ*micF* backgrounds. The specific activity of OppA-LacZ is decreased by 40% in the absence of MicF. For TcyJ-LacZ, a small 18% increase is observed (Fig. 1C).

Previous reports demonstrated that GcvB pairs near MicF predicted pairing site on *tcyJ* mRNA to achieve downregulation (Fig. S1C) [30]. We therefore hypothesized that MicF base pairing could be hindered by the presence of GcvB. We performed TcyJ-LacZ β-galactosidase assay in Δ*gcvB* and Δ*gcvB*Δ*micF* mutant backgrounds and observed a 72% increase of TcyJ-LacZ activity when *micF* is not expressed, suggesting that the *tcyJ* mRNA is a negative target of MicF (Fig. 1C). The activity of the YjbJ-LacZ fusion was not affected by the Δ*micF* mutation (Fig. 1C).

We decided to focus on the *oppA* mRNA, the first positively regulated mRNA identified in MicF targetome. To confirm the specific co-purification of the *oppA* mRNA with MicF, we performed MS2 affinity purification (MAP) using either MicF or a truncated *oppA* RNA as baits and analysed the purified RNAs in Northern blot assays (Fig. S1D, E). In both cases, MicF and *oppA*, co-purified. The *ompF* mRNA and sRNAs GcvB and SgrS served as positive or negative controls in these assays. The longer transcripts observed in the *oppA* panel are most probably the result of termination read-through from both *oppA* and *oppA*-MS2 constructs.

Even though direct interaction between MicF and the *oppA* mRNA is suggested by *in vivo* and *in silico* results, we considered the possibility that the regulatory effect could be indirect, via the leucine-responsive regulatory protein (Lrp). Transcription of *oppA* is well characterized as regulated by Lrp [59] and expression of *lrp* is negatively regulated at the post-transcriptional level by MicF [33,37]. Using a *lacZ* reporter gene fused to *oppA* promoter (refer to Fig. S3), we demonstrate that *oppA* promoter activity is not affected by the knock-out of *micF* gene (Fig. S1F). Moreover, we show that MicF retains its regulatory effect on OppA-LacZ in a Δ*lrp* background (Fig. S1G). These results confirm that MicF does not achieve regulation of *oppA* mRNA through regulation of *lrp*.

Recently, a study published by Vogel’s team and performed in *Salmonella enterica* describes a sRNA, OppX, deriving from *oppABCDF* 5ʹUTR [60]. The OppX sRNA is present in three different forms, depending on processing: OppX-L (410 nts), OppX-M (190 nts) and OppX-S (109 nts). The OppX sRNA was characterized as a MicF sRNA sponge, sequestering MicF to derepress *ompF* mRNA. Even though the binding site of MicF in the 5ʹUTR of *oppA* mRNA is well conserved in Enterobacteriaceae, our results obtained in *E. coli* clearly suggest a different story as we were unable to detect any stable RNA fragments deriving from *oppA* 5ʹUTR (Fig. S1H). Furthermore, our results are supported by an RIL-Seq study performed in *E. coli* [35] in which MicF is interacting with fragments of *oppA* that are not contained to the OppX boundaries reported in *S. enterica* (Fig. 1B, red lines). Therefore, in *E. coli*, MicF does not interact with a fragment deriving from the *oppA* mRNA but rather interacts with the *oppA* mRNA itself.

Moreover, the various results presented here, obtained in *E. coli*, do not correlate with the findings in *S. enterica*, suggesting different roles and mechanisms involving the MicF sRNA and the *oppA* 5ʹUTR in these closely related bacteria.

### MicF sRNA pairs far upstream within *oppA* 5’ UTR

Next, to synthesize the *oppA* transcript *in vitro*, we first sought to determine the transcription start site (TSS) of *oppA*. In 1997, Igarashi and colleagues identified three putative TSS for *oppA* in the polyamine-requiring mutant strain MA261 [61]. To confirm the presence of these TSSs in *E. coli* K-12 MG1655, we performed primer extension (PE) assays and observed two TSS identical to those identified by Igarashi and colleagues, P1 (+1) and P2 (+246) (Fig. S3A). P1 and P2 have also been identified in genome-wide mapping of TSS in *E. coli* [62]. The results of our assays, however, indicate that the third TSS identified by Igarashi and colleagues, P3 (+341) is actually an RNase E-dependent processing site (Fig. S3A, B). The data is also supported by the analysis of XRN-1-treated RNA (Fig. S3C). Finally, our *in vivo* data indicate that P1 leads to the strongest OppA-LacZ β-galactosidase activity (Fig. S3D, E) and that the transcript is then processed by RNase E at the identified cleavage site (Fig. S3AB).

**Figure 2.**
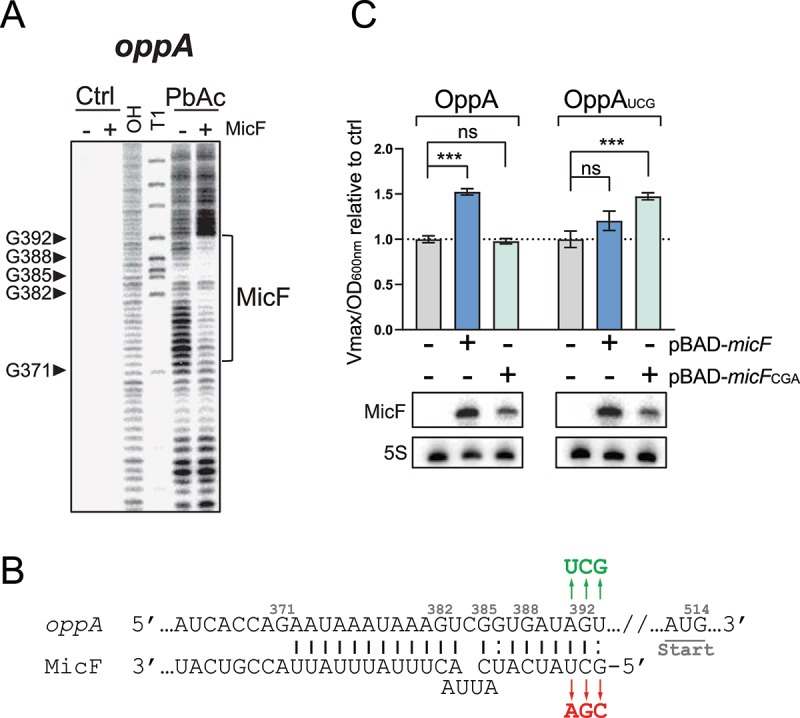
MicF regulates *oppA* through direct base pairing. **(A)** Lead acetate (PbAc) probing of γ-*oppA* with MicF. γ-*oppA* was incubated for 10 min with or without MicF (0.1 μM) prior to the addition of PbAc. Ctrl; non-reacted controls, OH; alkaline ladder, T1; and RNase T1 ladder. Numbers on the left indicate nucleotide position relative to P1 transcriptional start site (see Fig. 8A). The putative MicF pairing site is indicated with a bracket. Competing yeast tRNA was used at a concentration of 0.15 mg/ml. **(B)** Schematization of MicF pairing site on the *oppA* mRNA. Mutations introduced in MicF to obtain MicF_CGA_ are indicated in red. Compensatory mutations introduced in *oppA* to obtain *oppA*_UCG_ are indicated in green. **(C)** β-galactosidase assay of OppA-LacZ (left) or OppA_UCG_-LacZ (right). Expression of *micF* and *micF*_CGA_ was induced by the addition of 0.025% and 0.75% arabinose, respectively, at OD_600nm_ = 0.5. Samples (N = 3, mean ± SD) were taken at OD_600nm_ = 2.0. ***p = 0.0010, ns: *p* > 0.05, unpaired two-tailed Student’s *t* test. Representative Northern blots of MicF are shown. 5S rRNA was used as a loading control.

Using *in vitro* transcribed *oppA* RNA starting at the cleavage site (+341), we performed an *in* *vitro* probing assay with lead acetate (PbAc) in the absence or presence of MicF. We observed a clear protection of nucleotides +372 to +392 in the presence of MicF, suggesting a potential pairing site more than 100 nts upstream of *oppA* mRNA initiator codon (Fig. 2A, S4A). To validate that the protection is due to MicF base pairing to *oppA* mRNA, we introduced point mutations in MicF to obtain MicF_CGA_ (Fig. 2B). We performed electrophoretic mobility shift assays (EMSAs) of radiolabeled *oppA* RNA (γ-*oppA*) and results showed that MicF interacts with γ-*oppA*, but the shift observed with MicF_CGA_ required a higher concentration of sRNA (Fig. S5). We then introduced compensatory mutations in *oppA* to obtain *oppA*_UCG_ (Fig. 2B) and EMSAs were performed on γ-*oppA*_UCG_ in the presence of either MicF or MicF_CGA_. While MicF's capability to induce a shift of γ-*oppA*_UCG_ was greatly hindered, the complex formation was restored with MicF_CGA_ (Fig. S5). This suggests that the interaction between *oppA* mRNA and MicF is base pairing-dependent *in vitro*.

To assess if the MicF-dependent regulation of *oppA* mRNA observed previously (Fig. 1C) directly results from the base pairing between MicF and *oppA*, we overproduced either MicF or MicF_CGA_ in a strain carrying an OppA-LacZ fusion and performed β-galactosidase assays. Whereas the overexpression of MicF led to a 50% increase in OppA-LacZ activity, MicF_CGA_ had no effect (Fig. 2C). We then constructed the mutant OppA_UCG_-LacZ fusion, which restores base pairing to MicF_CGA_ (Fig. 2B). Although a slight regulation of OppA_UCG_-LacZ was observed when MicF was overproduced (20%), only the overproduction of MicF_CGA_ fully restored the upregulation of OppA_UCG_-LacZ (48%) (Fig. 2C). This demonstrates that the *in vivo* regulation of *oppA* mRNA by MicF depends on direct RNA–RNA interaction. Note that the induction of *micF* expression was performed with 0.025% and 0.75% arabinose for WT MicF and MicF_CGA_, respectively, to obtain more comparable levels of MicF and MicF_CGA_ sRNAs. One could argue that inducing WT MicF expression with higher arabinose concentration than 0.025% may activate translation of the OppA_UCG_-LacZ mutant construct due to massive MicF concentration. To assess if higher MicF concentration would lead to OppA_UCG_-LacZ regulation, we induced expression of *micF* with 0.1% arabinose (normal concentration) and observed regulation of WT OppA-LacZ but not of OppA_UCG_-LacZ, confirming the result obtained previously (Fig. S6).

### MicF activates *oppA* mRNA translation through a non-canonical mechanism of action

**Figure 3.**
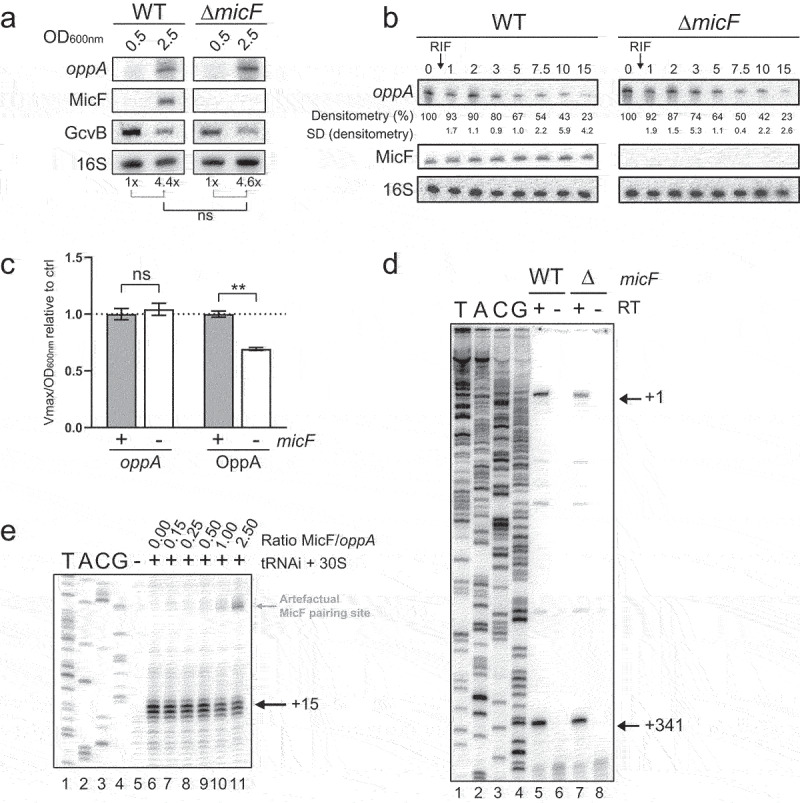
MicF regulates *oppA* at the translational level through a non-canonical mechanism. **(A)** Northern blot analysis of *oppA* mRNA in WT and Δ*micF* backgrounds. Samples were taken at 0.5 and 2.5 of OD_600nm_. 16S rRNA was used as a loading control. The data are representative of four independent experiments. **(B)** Half-life assays of *oppA*. 250 µg/ml Rifampicine was added at 2.0 OD_600nm_, after which samples were collected at specified time points. 16S rRNA was used as a loading control. The data are representative of four independent experiments. **(C)** β-galactosidase assay of OppA-LacZ translational and *oppA-lacZ* transcriptional, fusions in WT and Δ*micF*. Sample (N = 3, mean ± SD) were taken at OD_600nm_ = 3.7 and relativized to their respective WT conditions. **p = 0.0011, ns: *p* > 0.05, unpaired two-tailed Student’s *t* test. **(D)** Primer extension analysis of *oppA* mRNA extracted from WT and Δ*micF* strains. Lane 1–4: sequencing ladder. P1 (+1) and the cleavage site (+341) are indicated with arrows. **(E)** Toeprinting assay of γ-*oppA* in the presence of increasing concentration of MicF. Lane 1–4: sequencing ladder, lane 5: negative control. Annotation +15 represents the ribosome toeprint. The weak MicF pairing site is indicated in grey (see Figure S7B).

To characterize the mechanism by which MicF activates *oppA* mRNA translation, we monitored the effect of MicF on *oppA* mRNA at OD_600nm_ of 0.5 (exponential phase) and at 2.5 (stationary phase) by Northern blot (Fig. 3A). Analysis of four replicates confirmed that the knockout of *micF* does not affect *oppA* mRNA levels. Stability of *oppA* mRNA is also unaffected by *micF* knockout, as observed in half-life assays performed in WT and Δ*micF* backgrounds (Fig. 3B). We then constructed the *oppA-lacZ* transcriptional fusion and performed β-galactosidase assays in both WT and Δ*micF* backgrounds, using the translational fusion OppA-LacZ as a control. As expected, the activity of the transcriptional fusion remained stable (Fig. 3C, left), suggesting that MicF specifically activates *oppA* mRNA translation without affecting mRNA levels. By contrast, β-galactosidase activity of the translational fusion significantly decreased in the absence of MicF, indicative of positive regulation (Fig. 3C, right).

Our data identified an RNase E-dependent cleavage site in the 5ʹUTR of the *oppA* mRNA, at position +341 (Fig. S3). This cleavage site is located approximately 30 nucleotides upstream of the MicF pairing site (see Fig. 8A). We hypothesized that the pairing of MicF could modulate accessibility to the cleavage site, which could potentially lead to facilitated translation initiation of the unprocessed *oppA* mRNA. To determine if this was the case, we used total RNA extracted from the WT and Δ*micF* strains and performed primer extension assays. We observed no difference in the primer extension signals between WT and Δ*micF* RNAs, indicating that MicF does not modify the processing of *oppA* 5ʹUTR (Fig. 3D).

To assess whether MicF pairing to *oppA* mRNA increases binding of the 30S ribosomal subunit to the RBS of *oppA* RNA, we performed toeprinting assays. Using an *in vitro* transcribed *oppA* RNA starting at the cleavage site (+341) (Fig. S3), toeprinting was performed with increasing concentrations of MicF (Fig. 3E). Surprisingly, MicF did not facilitate binding of the 30S subunit and even acted as a weak inhibitor at higher ratios (lanes 10–11). Further investigations have led to the identification of a weak MicF base-pairing site, near the Shine–Dalgarno sequence of *oppA* mRNA, responsible for this competition at the RBS. However, this binding is only detectable at high concentration of MicF and in the absence of competing Yeast tRNA (Fig. S7). Considering the absence of MicF-dependent effect on the 30S subunit binding in toeprinting assays, our results suggest that MicF increases *oppA* mRNA translation through a mechanism independent of SD-sequestering structures.

### MicF activation of *oppA* translation depends on the negative regulation by GcvB sRNA

A previous report described the GcvB-dependent repression of the *oppA* mRNA [31]. GcvB has an extensive targetome composed of mRNAs encoding proteins involved in amino acid transport or biosynthesis [37]. It is strongly expressed in rich medium during the exponential growth phase as well as in the stationary phase [30]. GcvB is expressed in our experimental conditions and represses *oppA* mRNA (Fig. S1B).

**Figure 4.**
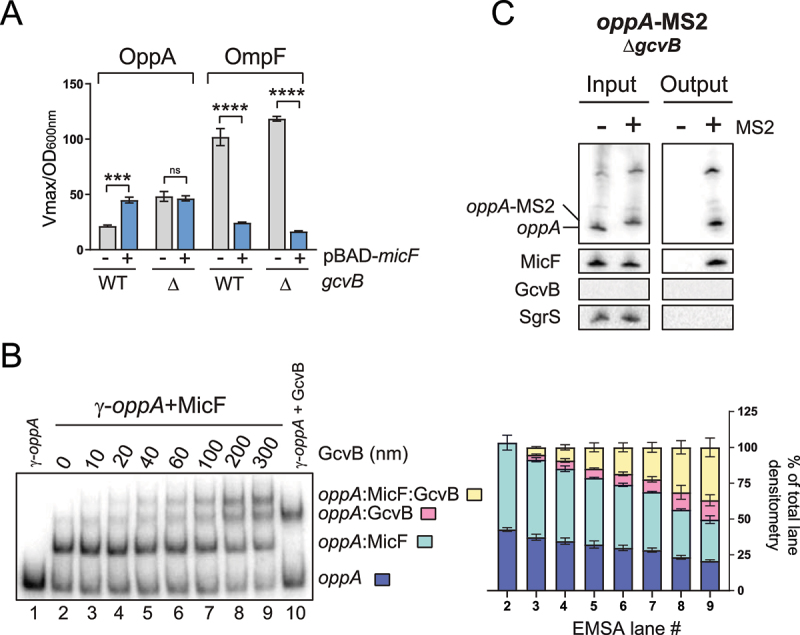
Regulation by GcvB is required to observe the MicF-dependent regulation of *oppA.* **(A)** β-galactosidase assay of OppA-LacZ (left) or OmpF-LacZ (right) in WT and Δ*gcvB* backgrounds. Expression of *micF* was induced by addition of 0.1% arabinose at OD_600nm_ = 0.5. Samples (N = 3, mean ± SD) were taken at OD_600nm_ = 2.0. ***p = 0.0001, ****p < 0.0001, ns: *p* > 0.05, unpaired two-tailed Student’s *t* test. **(B)** Left – Electrophoretic mobility shift assay of the pre-bound γ-*oppA*:MicF (5 nM: 300 nM, incubation 15 min at 37°C) complex incubated in the presence of increasing concentration of GcvB for 15 min at 37°C. γ-*oppA* (5 nM, lane 1), γ-*oppA*:MicF (5 nM: 300 nM, lane 2) and γ-*oppA*:GcvB (5 nM: 300 nM, lane 10) were used as controls to identify the different populations. Right – Densitometry analysis (N = 3, mean ± SD) of the different RNA complexes. **(C)** MS2 affinity purification of *oppA* and *oppA*-MS2 in a Δ*gcvB* background. Cells were harvested at OD_600nm_ = 2.0. SgrS sRNA served as a negative control. The results are representative of two independent experiments.

We hypothesized that an indirect interplay between MicF and GcvB could potentially dictate the regulation of the *oppA* mRNA. Both sRNAs interact at very distinct regions on the *oppA* mRNA: MicF pairs far upstream in the 5ʹUTR and GcvB interacts at the translation initiation region (Fig. 8A, S2). If both sRNAs antagonize each other following their pairing on the *oppA* mRNA, we would expect a stronger effect of MicF in a Δ*gcvB* background compared to a WT background. We therefore verified the effect of MicF on *oppA* mRNA in the absence of GcvB. We overexpressed MicF in Δ*micF* and Δ*micF*Δ*gcvB* strains harbouring the OppA-LacZ fusion or the OmpF-LacZ fusion, the latter being a positive control of the MicF activity (Fig. 4A). Surprisingly, the positive regulation of OppA-LacZ by MicF is completely lost in the Δ*gcvB* background. Regulation of the control fusion OmpF-LacZ by MicF was detected in the absence of GcvB, dismissing the possibility of the Δ*gcvB* mutation generally disrupting the ability of MicF to regulate mRNAs. Very similar results were obtained using the *gcvB*ΔR1 mutant (Fig. S8A), which lacks the region base pairing to the *oppA* mRNA [63]. This suggests that loss of the direct regulation of OppA-LacZ by GcvB causes the loss of MicF regulation.

These results can be explained if (i) MicF acts by sequestering GcvB, (ii) MicF prevents GcvB from interacting with *oppA* mRNA, or (iii) MicF is unable to interact with *oppA* mRNA in the Δ*gcvB* background. To address the first possibility, we used CopraRNA *in silico* predictor [36–38] for potential base pairing between MicF and GcvB. No putative interaction was detected between both sRNAs. Then, we sought a putative *in vivo* interaction between MicF and GcvB using the RIL-Seq data [35]. No interaction between GcvB and MicF was detected in all tested conditions (log phase, stationary phase and iron limitation). Finally, we looked at the enrichment of MicF in the MS2-GcvB MAPS data [30] and that of GcvB in the MS2-MicF MAPS data. Even though a few reads can be visualized on the BedGraph representation of the data (Fig. S8B), the number of reads, or the enrichment ratio of the MS2-sRNA/sRNA, was below our cut-offs. Moreover, GcvB seemed to bind non-specifically to either the chromatography column or to the MS2 aptamer, as suggested by the MS2 control experiment (Fig. S8C). However, this non-specific binding was not detected in Northern blots performed on affinity-purified samples (Fig. S1F). Overall, *in silico* predictions and *in vivo* experiments do not support a direct interaction between MicF and GcvB. To confirm that MicF does not act by titrating GcvB, we performed an EMSA of radiolabeled GcvB (γ-GcvB) with increasing concentration of MicF. No interaction between GcvB and MicF was detected. As a positive control, γ-GcvB was incubated with the *oppA* mRNA, and a clear shift was observed (Fig. S8D). Finally, the toeprinting assays confirmed that MicF does not interfere with GcvB-dependant regulation of *oppA* mRNA (Fig. S8E).

We next used EMSAs to verify if GcvB can interact with a MicF-bound *oppA* RNA molecule (Fig. 4B). γ-*oppA*, γ-*oppA*+MicF and γ-*oppA*+GcvB were used as controls to distinguish between the different shifted populations. Densitometry analysis of the EMSA shows that GcvB interacts with γ-*oppA* and with the γ-*oppA*-MicF complex (Fig. 4B, right panel). Furthermore, the knockout of *gcvB* gene results in the upregulation OppA-LacZ in a WT or a Δ*micF* background (Fig. S8F). Finally, the *oppA*-MS2 construct was used to validate whether MicF could still be co-purified with *oppA* in a Δ*gcvB* background. The results show that even in the absence of GcvB, MicF still co-purifies with the *oppA* mRNA, indicating that the Δ*gcvB* mutation does not hinder base pairing of MicF to the *oppA* mRNA (Fig. 4C). These results suggest that MicF does not act on the *oppA* mRNA translation by competing with the GcvB-dependent regulation.

**Figure 5.**
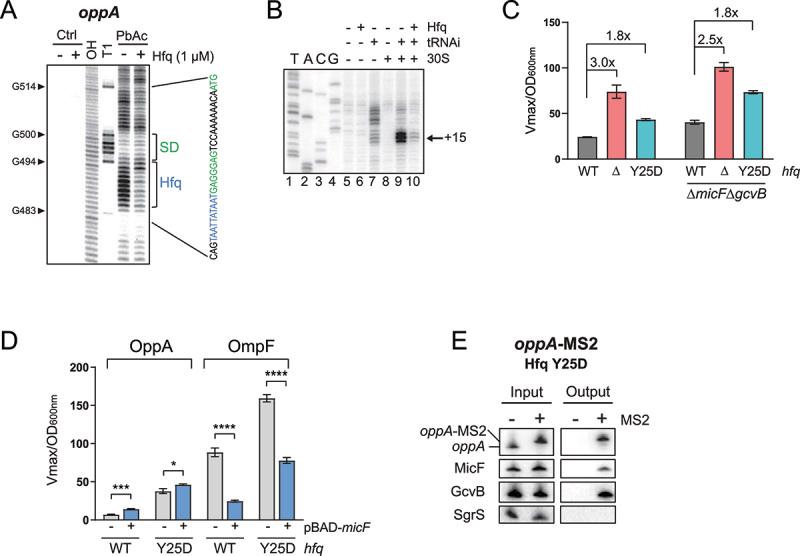
The Hfq chaperone is a negative regulator of *oppA* translation. **(A)** Footprinting analysis of γ-*oppA* in presence or absence of 1 μM purified Hfq protein. Ctrl; non-reacted controls, OH; alkaline ladder, T1; RNase T1 ladder. Numbers on the left indicate nucleotide position relative to the P1 transcriptional start site. The Hfq binding site and the Shine–Dalgarno (SD) are indicated with brackets in blue and green, respectively. Competing yeast tRNA was used at a concentration of 0.15 mg/ml. **(B)** Toeprinting assay of γ-*oppA* in presence or absence of purified Hfq protein (1 μM). Lane 1–4: sequencing ladder; lane 5–8: negative controls. Position +15 from the start codon is indicated by an arrow. Annotation +15 represents the ribosome toeprint. **(C)** β-galactosidase assay of OppA-LacZ translational fusion in three *hfq* backgrounds (WT, Δ*hfq, hfq* Y25D) and in two sRNA backgrounds (WT or Δ*micF*Δ*gcvB*). Samples (N = 2, mean ± SD) were taken at OD_600nm_ = 2.0. Fold changes are indicated. **(D)** β-galactosidase assay of OppA-LacZ (left) or OmpF-LacZ (right) in Δ*micF* and Δ*micF/hfq* Y25D backgrounds. Expression of *micF* was induced by addition of 0.1% arabinose at OD_600nm_ = 0.5. Samples (N = 3, mean ± SD) were taken at OD_600nm_ = 2.0. The data were relativized to the pNM12 empty vector control for each condition. *p = 0.02, ***p = 0.0001, ****p < 0.0001, ns: *p* > 0.05, unpaired two-tailed Student’s *t* test. **(E)** MS2 affinity purification of *oppA* and *oppA*-MS2 in a *hfq* Y25D background. Cells were harvested at OD_600nm_ = 2.0. GcvB and SgrS sRNAs served as positive and negative controls, respectively. The results are representative of two independent experiments.

### Hfq directly regulates *oppA* mRNA and is indirectly required for MicF-dependent positive regulation of *oppA* mRNA

Hfq is a major RNA chaperone that also acts as a direct regulator of translation [64–67]. This hexamer protein harbours multiple RNA binding sites, mainly the proximal and distal faces, preferring a poly(U) region or an (A-R-N)_n_ motif, respectively [68,69]. The rim of Hfq is also able to interact with RNA, mostly through AU-rich regions. Since the distal face is generally involved in interaction with mRNAs [69–71], we sought (A-R-N)_n_-like motifs in the 5ʹUTR of *oppA* that could serve as binding sites for the distal face of Hfq. We identified a putative Hfq binding region at position +484 to +492, near the SD. Using an *in vitro* footprinting assay, we confirmed that this region is protected in the presence of Hfq (Fig. 5A, 8A, S4B). We then asked if Hfq binding could directly regulate *oppA* mRNA in the absence of sRNA partners. In a toeprinting assay, we observed a clear decrease in 30S ribosomal subunit binding to *oppA* mRNA in the presence of Hfq, suggesting that Hfq alone blocks *oppA* mRNA translation initiation *in vitro* (Fig. 5B).

**Figure 6.**
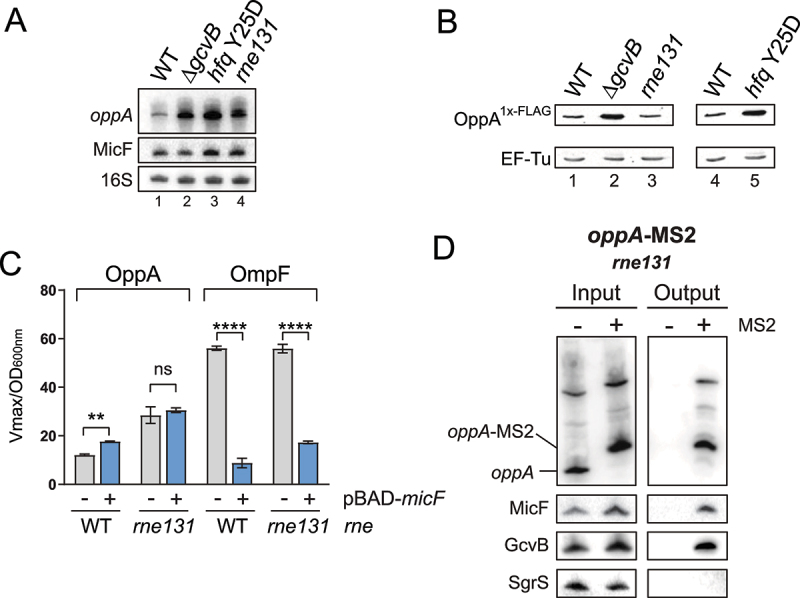
MicF-dependent regulation of *oppA* depends on *oppA* cellular mRNA concentration. **(A)** Northern blot analysis of *oppA* and MicF in WT, Δ*gcvB, hfq* Y25D and *rne131* backgrounds. Samples were taken at OD_600nm_ = 2.0. 16S rRNA was used as a loading control. The data are representative of two independent experiments. **(B)** Western blot analysis of the OppA^1x-^^FLAG^ protein in WT, Δ*gcvB, rne131*, or *hfq* Y25D backgrounds. EF-Tu was used as a loading control. The data are representative of two independent experiments. **(C)** β-galactosidase assay of OppA-LacZ (left) and OmpF-LacZ (right) in Δ*micF* and Δ*micF/rne131* backgrounds. Expression of *micF* was induced by addition of 0.1% arabinose at OD_600nm_ = 0.5. Samples (N = 3, mean ± SD) were taken at OD_600nm_ = 2.0. **p = 0.0029, ****p < 0.0001, ns: *p* > 0.05, unpaired two-tailed Student’s *t* test. **(D)** MS2 affinity purification of *oppA* and *oppA*-MS2 in a *rne131* background. Cells were harvested at OD_600nm_ = 2.0. GcvB and SgrS sRNAs served as positive and negative controls, respectively. The results are representative of two independent experiments.

To assess the role of Hfq in *oppA* mRNA translational regulation *in vivo*, we performed β-galactosidase assays of OppA-LacZ in backgrounds addressing the role of Hfq (WT, Δ*hfq* and *hfq* Y25D), in two sRNA backgrounds (WT and Δ*micF*Δ*gcvB*). The Y25D mutation in the distal face of Hfq alters its binding to AU-rich sequences, such as the Hfq binding site in the *oppA* mRNA but does not affect binding to class 1 sRNAs, such as MicF and GcvB [72]. We observed a significant increase in β-galactosidase activity in the Δ*hfq* and in the *hfq* Y25D backgrounds when compared to the WT (Fig. 5C, left). This indicates that the absence of Hfq, or its inability to interact with the *oppA* mRNA, alleviates translational repression of the mRNA. Similar regulation ratios were observed in the Δ*micF*Δ*gcvB* background, suggesting that Hfq regulates the *oppA* mRNA independently of the known sRNAs interacting with the *oppA* mRNA (Fig. 5C, right).

We then asked if hindering Hfq binding to *oppA* mRNA would affect the MicF-dependent regulation *in vivo*. We opted to test the effect of MicF overproduction on both the OppA-LacZ and OmpF-LacZ fusions in Δ*micF* and Δ*micF*/*hfq* Y25D backgrounds. We favoured the use of Hfq Y25D over a Δ*hfq* strain because the Y25D mutation does not decrease MicF (Fig. 6A) or GcvB [73] steady-state levels. Whereas MicF-dependent regulation of OppA-LacZ was partially lost in the *hfq* Y25D strains, regulation of OmpF-LacZ remained (Fig. 5D, S9). We then used *oppA*-MS2 MAP followed by Northern blot analysis to show that MicF still pairs with *oppA* mRNA in the *hfq* Y25D background (Fig. 5E). Thus, MicF-dependent regulation of the *oppA* mRNA seems to depend on the repression of translation by Hfq.

**Figure 7.**
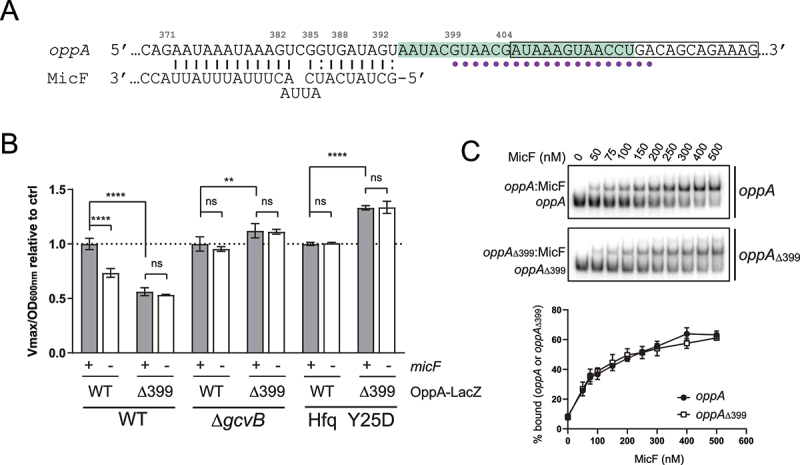
MicF potentially regulates *oppA* through a translational enhancer. **(A)** Schematic representation of a portion of *oppA* 5ʹUTR. Pairing site of MicF is shown. In green: region more accessible to PbAc cleavage in the presence of MicF (Fig. S4A). Boxed: region comprised in the Ribo-seq peak (Fig. S10). Nucleotides deleted in *oppA*_Δ399_ are indicated by a dot. Nucleotides are numbered relative to P1. **(B)** β-galactosidase assay of OppA-LacZ and OppA_Δ399_-LacZ in WT and Δ*micF*, in Δ*gcvB* and Δ*gcvB*Δ*micF*, or in *hfq* Y25D and *hfq* Y25D/Δ*micF* backgrounds. Samples (N = 3, mean ± SD) were taken at OD_600nm_ = 2.0. Data of each Δ*micF* condition (white bars) were relativized to their corresponding WT *micF* condition (grey bars). **p = 0.0089, ****p < 0.0001, ns: *p* > 0.05, two-way ANOVA, multiple-comparison test. **(C)** Electrophoretic mobility shift assay of γ-*oppA* or γ-*oppA*_Δ399_ incubated in the presence of increasing concentration of MicF for 15 min. Below, densitometry analysis is plotted as the fraction of γ-*oppA* or γ-*oppA*_Δ399_ bound to MicF. The data are representative of two independent experiments.

### MicF-dependent regulation of *oppA* depends on the mRNA cellular concentration

Previously, we showed that two negative regulators of *oppA* mRNA, GcvB and Hfq, are individually essential for the MicF-dependent activation of *oppA* mRNA. We therefore sought to determine a common characteristic shared by the Δ*gcvB* and *hfq* Y25D mutants. Northern blot analyses show increased levels of *oppA* mRNA in both backgrounds compared to WT (Fig. 6A, lanes 1 to 3). Moreover, both Δ*gcvB* and *hfq* Y25D strains present higher levels of the OppA protein, as shown by Western blot (Fig. 6B, lanes 1, 2 and 4, 5).

We then determined if the increase in *oppA* mRNA or OppA protein levels caused the loss of MicF-dependent regulation. We observed that the *rne131* mutation causes an accumulation of the *oppA* mRNA transcript (Fig. 6A, lanes 1, 4) without affecting OppA protein levels (Fig. 6B, lanes 1, 3). Therefore, using the *rne131* mutant, we assessed the impact of increased *oppA* mRNA levels on the MicF-dependent regulation of *oppA* mRNA. We overproduced MicF and monitored the activity of the OppA-LacZ fusion in Δ*micF* and Δ*micF*/*rne131* backgrounds. The overproduction of MicF did not affect the activity of the OppA-LacZ fusion in the *rne131* background (Fig. 6C). To confirm that MicF could still pair to the *oppA* mRNA in *rne131* strains, we performed Northern blot on MAP samples. The results show specific enrichment of MicF and GcvB with *oppA*-MS2, demonstrating that the *rne131* allele does not affect the pairing of these sRNAs to *oppA* mRNA (Fig. 6D). Moreover, we confirmed previously that the *rne131* mutation did not affect GcvB-dependent regulation of the *oppA* mRNA (Fig. S1B). These results suggest that MicF can regulate the *oppA* mRNA only when the *oppA* mRNA is expressed below a certain level.

### MicF potentially upregulates the *oppA* mRNA by modulating accessibility to a translation-enhancing region

Our data suggest that MicF positively regulates *oppA* mRNA translation by an unknown mechanism. Among the cis-encoded mRNA elements involved in modulation of translation initiation are the translational enhancers (TEs). TEs are usually AU- or CA-rich sequences located upstream or downstream of the SD sequence that facilitate translation initiation [74–76]. Based on our probing assay of *oppA* mRNA, we noticed a region seemingly more sensitive to cleavage in the presence of MicF. This AU-rich stretch of 23 nts is located immediately downstream of the MicF pairing site (Fig. 7A, Fig. S4A). We asked if this region of the *oppA* mRNA could act as a TE whose accessibility depends on the pairing of MicF. Ribosome profiling data obtained from our lab (Geffroy *et al.*, in preparation) indicated a modest but clearly defined peak corresponding to the AU-rich stretch located immediately downstream of the MicF base-pairing site (Fig. 7A, S10).

**Figure 8.**
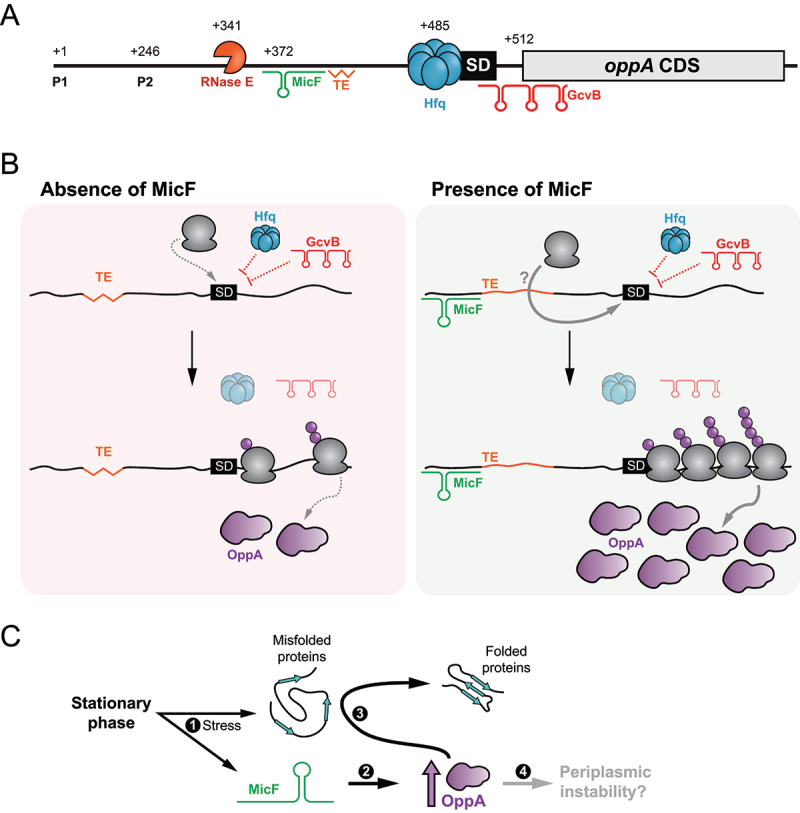
Schematic representation of the post-transcriptional regulation of *oppA* expression. **(A)** Scheme of *oppA* mRNA. Transcriptions can originate from both transcription start sites (P1 and P2). The mRNA is cleaved in its 5ʹUTR by the RNase E endonuclease at position +341. The Hfq protein binds just upstream of the SD to impede translation. The GcvB sRNA binds the RBS to hinder translation. MicF positively regulates *oppA* translation by base pairing at positions +372 to +393. A putative translational enhancer is located immediately downstream of the MicF base-pairing site. The numbers indicate the nucleotide position relative to P1. **(B)** Proposed mechanism of *oppA* regulation. Left – in the absence of MicF, the TE is hardly accessible (represented by a jagged line). Hfq and GcvB repress translation of *oppA*. Translation occurs at a minimal rate. Right – in the presence of MicF, the sRNA renders the TE more accessible (regular line) and favours translation through an unknown mechanism directly involving the ribosome or not. Even in the presence of Hfq and GcvB, which hinder translation, the now accessible TE allows efficient translation of *oppA*. **(C)** Proposed physiological role of the MicF-dependent regulation of *oppA*. 1 – The stationary phase of growth causes membrane stress. MicF is expressed and misfolded proteins start accumulating in the periplasm. 2 – MicF increases the translation of *oppA* to a specific threshold. 3 – OppA can act as a chaperone in the periplasm, facilitating protein folding. 4 – An excessive increase in OppA protein levels could lead to periplasmic instability.

To address the potential role of this mRNA region as a TE, we designed a deletion mutant where nucleotides deleted to generate *oppA*_Δ399_ are indicated in Fig. 7A. *In silico* structure predictions are presented in Supplementary Figure S11. β-Galactosidase assays were performed on OppA_Δ399_-LacZ in WT and Δ*micF* strains. First, we observed a decrease of 46% for OppA_Δ399_-LacZ β-galactosidase activity compared to OppA-LacZ (Fig. 7B, left, grey bars). This suggests that deleting the AU-rich region between nucleotides 399 and 413 of the *oppA* mRNA negatively impacts translation, supporting the idea of this 5ʹUTR region acting as a translational enhancer. Furthermore, a complete loss of the MicF-dependent regulation was observed for OppA_Δ399_-LacZ (Fig. 7B, left). To exclude the possibility of MicF being unable to interact with *oppA*_Δ399_, we performed EMSAs in the presence of an increasing concentration of MicF (Fig. 7C, top). The results show that the *oppA*_Δ399_ mutant interacts similarly with MicF than the WT *oppA* (Fig. 7C, bottom).

Since the MicF-dependent regulation of *oppA* mRNA is inhibited by the Δ*gcvB* mutation or by the *hfq* Y25D mutation, and that the function of the TE seems to be intimately linked to that of MicF, we asked whether the deletion of the TE would affect β-galactosidase activity in those mutants. Thus, we performed β-galactosidase assays of the OppA-LacZ and OppA_Δ399_-LacZ fusions in the Δ*gcvB* and *hfq* Y25D backgrounds. After deleting the *oppA* mRNA TE, no decrease of translational activity occurs in the absence of GcvB (Fig. 7B, middle, grey bars) or in the *hfq* Y25D mutant (Fig. 7B, right, grey bars). Moreover, as expected, the Δ*micF* mutation has no effect in both mutant backgrounds Δ*gcvB* and *hfq* Y25D (Fig. 7B, middle and right). These results support a model in which the MicF-dependent regulation of *oppA* mRNA can occur through the modulation of a TE element, at low *oppA* mRNA cellular concentrations.

### Discussion

In the present work, a combination of *in vivo* and *in silico* techniques led to the identification of two new targets of MicF, the *tcyJ* and *oppA* mRNAs. While the *tcyJ* mRNA is the fifth negative target of MicF, the *oppA* mRNA is its first positively regulated target. We demonstrate that the pairing of MicF in the 5ʹUTR of the *oppA* mRNA promotes mRNA translation without affecting transcript stability.

The regulatory effect of MicF on *oppA* mRNA translation does not appear to be conserved between closely related species, even though the interaction between the two entities is conserved. Indeed, Vogel’s team recently identified an sRNA deriving from *Salmonella enterica oppA* 5ʹUTR that acts as a sponge RNA to sequester MicF. In *E. coli*, however, two major differences must be noted. First, no stable RNA fragment originating from *oppA* 5ʹUTR could be detected. Second, we clearly demonstrate that MicF is a true regulator of *oppA* mRNA translation. Here, we propose that MicF can positively regulate *oppA* mRNA translation by modulating the accessibility of a translational enhancer located approximately 100 nts upstream of *oppA* RBS. To date, this major difference between the roles of MicF and of the *oppA* mRNA 5ʹUTR in *S. enterica* and *E. coli* cannot be explained. However, this would not be the first instance of differential regulation of gene expression between these two closely related bacteria. This is not surprising considering the different environmental pressures dictating the evolution of *S. enterica* and *E. coli*.

Many mechanisms of positive regulation have been previously described, with two of them standing out. First, sRNAs are known to base-pair in the 5ʹUTR of their target to disrupt inhibitory structures, resulting in higher translation rates. This mechanism was first uncovered in the Gram-positive bacterium *Staphylococcus aureus*, in which RNAIII upregulates *hla* [77]. Since then, many sRNAs have been shown to employ a similar mechanism, as it is the case for the regulation of *shiA* by the sRNA RyhB [48]. In the pathogen *Listeria monocytogenes*, the pairing of sRNA Rli27 far upstream of the RBS (−151 to −129 relative to the start codon) is hypothesized to modify the structure of the entire 5ʹUTR, liberating the RBS and increasing translation initiation efficiency [78]. This last example is reminiscent of MicF pairing 120 nts upstream of *oppA* start codon. However, *in vitro* structural probing of *oppA* RNA showed no disruption of mRNA secondary structures near the RBS. Additionally, we demonstrated that MicF is unable to promote binding of the 30S ribosomal subunit to *oppA* RNA *in vitro*.

In the second mechanism of positive regulation, sRNAs have previously been shown to interfere with mRNA decay, mainly by masking endonuclease cleavage sites. In *E. coli*, the SgrS sRNA positively affects the *yigL* mRNA by base pairing in the coding sequence masking an RNase E cleavage site and stabilizing the mRNA [79]. Contrasting with this example, MicF knockout or overproduction has no influence on the *oppA* mRNA levels or stability, ruling out the possibility of MicF interfering with RNases cleavage sites. Furthermore, sRNAs are known to be able to promote or obstruct the processing of their targets [80], but this explanation was rapidly dismissed in the present case, based on the analysis of *oppA* 5ʹUTR processing.

The roles of other cis-encoded elements of the *oppA* mRNA were considered throughout our investigation of the MicF-dependent regulation of *oppA* mRNA (Fig. S2). Polyamines, such as spermidine, have been shown to promote *oppA* mRNA translation through a riboswitch located in the mRNA 5ʹUTR (Fig. S2) [61,81,82]. While MicF could participate in riboswitch regulation, several data point otherwise. Indeed, the MicF pairs approximately 60 nts upstream of the riboswitch, limiting the possibility of direct interference. Moreover, no structural modification, apart from the TE region located immediately downstream of the MicF pairing site, was observed in *oppA* 5ʹUTR following MicF pairing.

In *E. coli*, an *oppA* deletion mutant is more sensitive to heat shock compared to a wild-type strain, suggesting a role for OppA in the heat shock response [83]. A variation in temperature could therefore increase, or impair, MicF capability to regulate *oppA* mRNA through structural modifications. However, the regulation observed here is at constant temperature, suggesting that the MicF-dependent regulation of *oppA* does not depend on temperature-modulated modifications of *oppA* 5ʹUTR structure.

We therefore explored the trans elements involved in *oppA* mRNA expression. The *oppA* mRNA has previously been shown as a direct negative target of the GcvB trans-acting sRNA [29]. In our experimental conditions, the loss of GcvB expression (Δ*gcvB*) causes the loss of MicF-dependent regulation even though no interaction or indirect competition between both sRNAs was detected. The dual regulation of the *oppA* mRNA by both MicF and GcvB is especially intriguing. There are only a few examples of mRNAs that are both positively and negatively regulated by sRNAs. The first case of antagonistic regulation concerns *flhD*, which is negatively regulated by ArcZ, OmrA, OmrB and OxyS [84] and positively regulated by McaS [85]. Another well-characterized case is *rpo*S mRNA, which is upregulated by DsrA, ArcZ and RprA [86–88] and downregulated by CyaR [89,90]. In both situations, though, three regulators or more act in concert versus a single antagonistic regulator, suggesting that only a highly specific set of environmental conditions would lead to a switch in regulation of the target. Moreover, both sRNAs McaS and ArcZ share a base-pairing site on *flhD* target, creating competition between both sRNAs [84,85]. As for the regulation of *rpoS*, the sRNA ArcZ specifically pairs and promotes degradation of the CyaR, creating an exclusive regulation: CyaR can only regulate *rpoS* in the absence of ArcZ [90]. Finally, the *cirA* mRNA is positively regulated by RyhB and negatively by OmrA and OmrB sRNAs [91–93]. While RyhB is mainly expressed during iron-starvation conditions [94], OmrA/B are mostly expressed in high osmolarity conditions [92]. Again, only a specific set of conditions would cause expression of RyhB and OmrA/B at the same time.

The dual regulation of *flhD, rpoS* and *cirA* target mRNAs contrasts with that of the *oppA* mRNA by MicF and GcvB sRNAs. Indeed, MicF and GcvB are concurrently expressed during the early stationary phase of growth (experimental condition of this study), and they pair at distinct sites on the *oppA* mRNA. Moreover, both MicF and GcvB can accumulate without promoting each other’s degradation. Our initial results suggest that GcvB repression is a prerequisite to observe the positive regulation of *oppA* mRNA by MicF. Further investigation revealed that a combination of GcvB, Hfq and the RNA degradosome complex reduces *oppA* mRNA expression to enable translational activation by MicF.

Previously, Hfq was shown to directly regulate specific mRNA translation. For example, it directly binds the RBS of the IS10 transposase mRNA to block translation initiation [65], interacts with cis-encoded elements of the *cirA* mRNA to modulate translation [91]. More recently, a study found that the binding of Hfq in the 5ʹUTR of the *mutS* mRNA causes remodelling of the secondary structure, hindering *mutS* translation [66]. Here, we demonstrate that Hfq interacts with *oppA* mRNA in the translation initiation region, just upstream of the RBS to prevent 30S ribosomal subunit binding to the mRNA, probably through steric inhibition. Like GcvB, the presence of Hfq is required to observe the MicF-dependent regulation of *oppA* mRNA. Taken together, these results indicate that MicF can only regulate *oppA* mRNA in a narrow range of cellular mRNA levels, which is made possible by the concerted action of GcvB, Hfq and RNase E. This is highly reminiscent of the regulation of RyhB by the 3’ external transcribed spacer of the *glyW-cysT-leuZ* pre-tRNA transcript (3ʹETS*^leuZ^*), which is only capable of exerting its effect on RyhB at low cellular RyhB levels [19].

Investigation of the *oppA* mRNA sequence revealed a putative translational enhancer rendered more accessible by the presence of MicF. We were able to show that this region of *oppA* mRNA was important to achieve optimal translation rates. More importantly, we presented evidence that MicF might act by modulating the access of this putative TE site to promote translation initiation. This positive mechanism of regulation is surprising as it contrasts with other examples of sRNAs interacting with TEs. Indeed, the GcvB sRNA represses translation of both *gltI* and *dppA* mRNAs by masking CA-rich TEs, and SgrS sRNA obstructs the U-rich TE of *manX* mRNA to hinder translation [95]. However, the mechanisms involved in TE-dependent translation modulation are not yet fully understood. In some cases, the S1 ribosomal protein contacts the TE and stabilizes the ribosome at the translation initiation site. The S1 protein can also contact TEs to help promote initiating ribosomes to the elongation step [76].

The S1 protein, the largest of the ribosomal proteins, is composed of six domains. While domains 1 to 4 are essential for cell viability [96], truncation of domains 5 and 6 (*rpsA*Δ56) results in a viable strain. Although their role is not yet fully understood, deletion of domains 5 and 6 in *E. coli* presents a strong growth defect. Domain 5 is highly similar to domain 4 and might be involved in RNA binding [96–98]. We asked whether the putative *oppA* TE we identified was S1-dependent. If this is the case, truncation of both domains 5 and 6 could affect the binding of S1 to *oppA* mRNA, and we would expect a reduced effect of MicF. Thus, we performed β-galactosidase assays of OppA-LacZ in WT or *rpsA*Δ56 backgrounds following the overexpression of MicF. Whereas the translational activity of *oppA* strongly decreases in *rpsA*Δ56 background, we observed no difference in the MicF regulation of *oppA* mRNA compared to WT (Fig. S10B). While the data presented here do not reveal the mechanism by which TE increases *oppA* mRNA translation, evidence demonstrate that *oppA* TE is fully functional when assisted by MicF (Fig. 8B).

MicF-dependent activation of *oppA* mRNA in the stationary phase of growth, while GcvB is active, seems to be counter-intuitive. OppA is the SBP of the oligopeptide (Opp) ABC transporter, responsible for the import of charged tripeptides and tetrapeptides [99–101]. The Opp ABC transporter is especially useful in conditions of amino acid starvation, a stress present in the late stationary phase of growth or in minimal medium. GcvB, however, is highly expressed in rich medium, and repressed when amino acids are scarce, alleviating the repression of many transporters, such as the Opp ABC transporter, the cytidine ABC transporter [30], the dipeptide (Dpp) ABC transporter [31], or the D-serine/alanine/glycine/-H^+^ symporter [102]. Therefore, why would MicF activate OppA when GcvB is still highly synthesized in the cells? We hypothesize that the answer could reside in the role of OppA as a periplasmic chaperone [6,7]. During the stationary phase of growth, an abundance of stresses, including the accumulation of misfolded proteins in the periplasm, induces the expression of the envelope stress response (ESR) effectors, especially the transcriptional factors σ^S^ and σ^E^ [103,104]. In such stressful conditions, σ^S^ triggers MicF synthesis, which helps upregulate OppA levels. OppA could then act as a periplasmic chaperone, preventing protein denaturation in the periplasm (Fig. 8C).

Previous work performed on the uropathogenic *E. coli* (UPEC) strain indicates that OppA is essential during infection [105]. This has been attributed to the central role of OppA in peptide uptake, possibly the main source of carbon for bacterial cells during urinary tract infections. Later, OppA was shown to be involved in the sensitivity to antibiotics in UPEC, particularly Polymyxin B [106]. Other toxic compounds, such as the synthetic tri-peptide tri-L-ornithine [107] or the recently developed antibiotic GE81112 [108], also rely on the presence of OppA to enter the cell. The second target of MicF we identified in this study, the *tcyJ* mRNA, also encodes a periplasmic substrate-binding protein, the cystine ABC transporter. Like the Opp ABC transporter, the cystine ABC transporter is involved in the uptake of the toxic analogues of two amino acids, L-selenaproline (SCA) and L-selenocystine (SeCys) [109]. Further experiments are needed to assess the role of MicF in antibiotic sensitivity in relation to the regulation of *oppA* and *tcyJ* mRNAs.

## Supplementary Material

Supplemental Material

## Data Availability

Galaxy is an open source, community-driven and web-based platform accessible at https://usegalaxy.org/ and can be found in a GitHub repository (https://github.com/galaxyproject). The UCSC Microbial Genome Browser is a web-based genome browser hosted by the University of California, Santa Cruz (http://microbes.ucsc.edu/). MicF MAPS data has been deposited to the NCBI Gene Expression Omnibus (GEO) under the accession number GSE113584 (token for reviewers: mpifekaarjsrpsx). MS2-control MAPS data is available on the NCBI Gene Expression Omnibus (GEO) under the accession number GSE67606.
