# Supplementary material for: Hfq protein and GcvB small RNA tailoring of oppA target mRNA to levels allowing translation activation by MicF small RNA in Escherichia coli
Source: RNA Biol. 2023 Mar 1;20(1):59–76. doi: 10.1080/15476286.2023.2179582 (PMC9988348; doi:10.1080/15476286.2023.2179582)
Supplement: Supplemental Material [file KRNB_A_2179582_SM2059.zip › sup file.docx]

**Supplementary Data for**

**Hfq Protein and GcvB Small RNA Tailoring of *oppA* Target mRNA to Levels Allowing Translation Activation by MicF Small RNA in *Escherichia coli***

Marie-Claude Carrier, David Lalaouna, Eric Massé

Corresponding author: Eric Massé

Email: eric.masse@usherbrooke.ca

**This file includes:**

Supplementary Figures S1 to S11


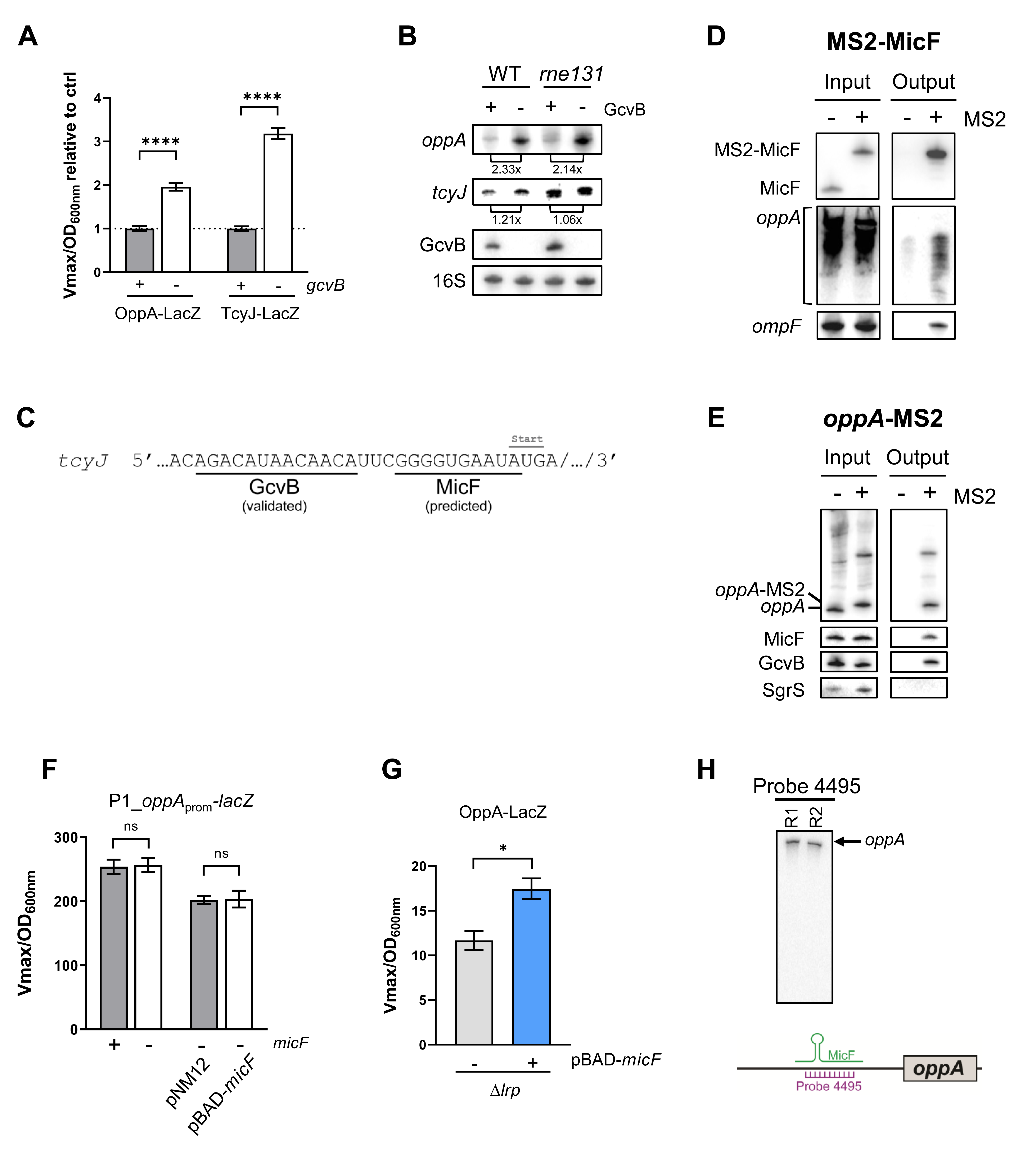


**Figure S1. (A)** β-galactosidase assay of OppA-LacZ or TcyJ-LacZ translation fusions in WT and Δ*gcvB* backgrounds. Samples (N=3, mean ± SD) were taken at OD_600nm_=2.0. *****P* < 0.0001, unpaired two-tailed Student’s *t* test. **(B)** Northern blot analysis of *oppA* and *tcyJ* mRNAs in presence or absence of GcvB in WT *rne* and *rne131* backgrounds. Samples (N=2) were taken at OD_600nm_=2.0. 16S rRNA was used as a loading control. Fold-change based on densitometry analysis is indicated below the panels. **(C)** Schematic representation of *tcyJ* translation initiation region. Validated pairing site of GcvB and predicted pairing site of MicF are indicated. **(D)** MS2 affinity purification of MicF or MS2-MicF. Constructs were overexpressed with 0.1% arabinose for 10 min at OD_600nm_=1.7. OmpF served as a positive control. N=2. **(E)** MS2 affinity purification of *oppA* or *oppA*-MS2. Cells were harvested at OD_600nm_=2.0. GcvB and SgrS sRNAs were used as positive and negative controls, respectively. N=2. **(F)** β-galactosidase assay of P1_*oppA­*-*lacZ* promoter fusion in WT and Δ*micF* backgrounds (left) or when MicF was overproduced from the pBAD-*micF* vector (right). When necessary, expression of *micF* was induced by addition of 0.1% arabinose at OD_600nm_=0.5. Samples (N=3, mean ± SD) were taken at OD_600nm_=2.0. ns: *P*> 0.05. **(G)** β-galactosidase assay of OppA-LacZ translation fusion in WT and Δ*lrp* backgrounds. Samples (N=3, mean ± SD) were taken at OD_600nm_=2.0. **P*= 0.0102, unpaired two-tailed Student’s *t* test. (H) Northern blot analysis of *oppA* visualized using a probe located at the pairing site of MicF, as depicted in the scheme (bottom). Two replicates (R1 and R2) are show. Samples (N=2) were taken at OD_600nm_=2.0.


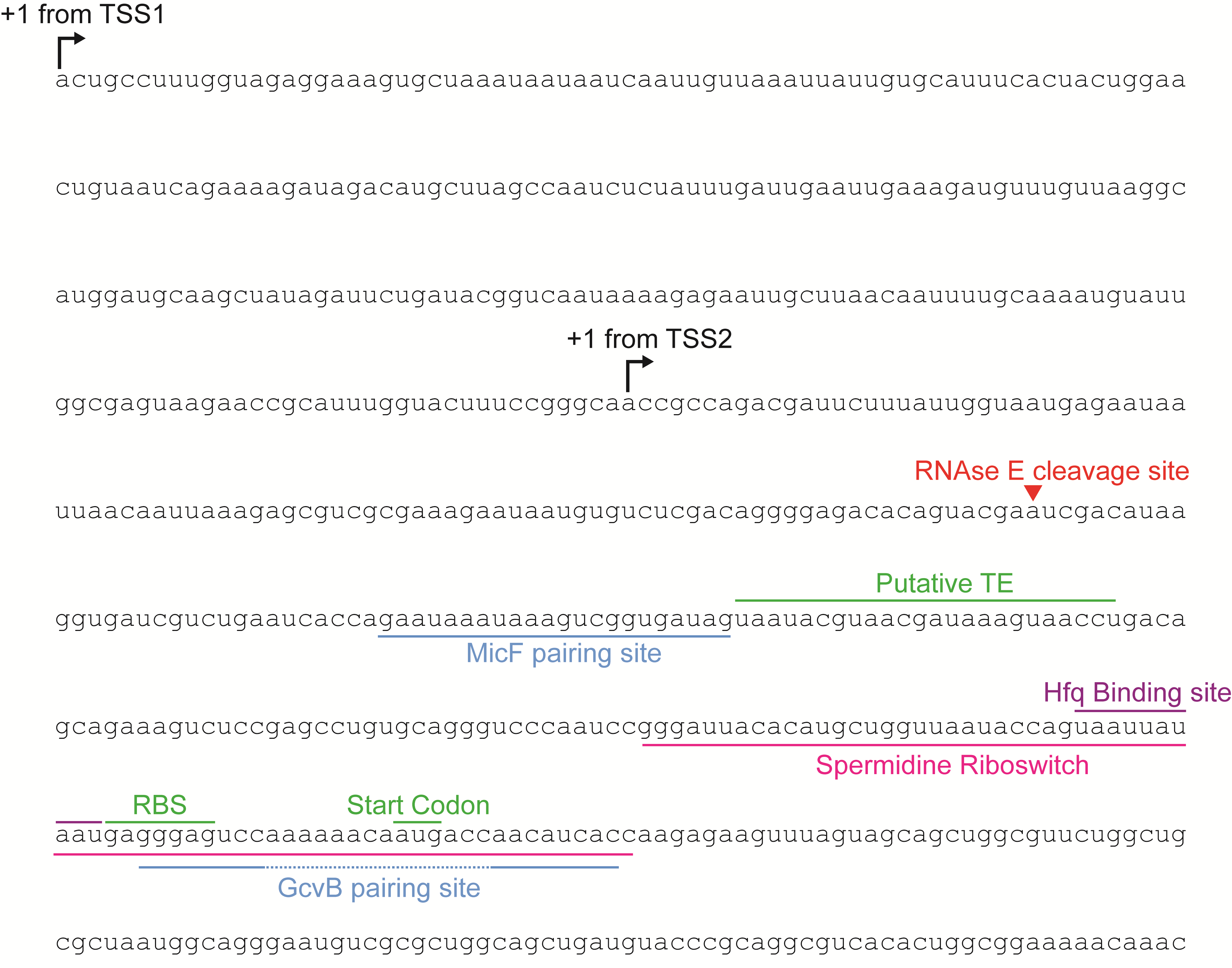


**Figure S2.** Sequence panel of *oppA* mRNA 5’UTR.


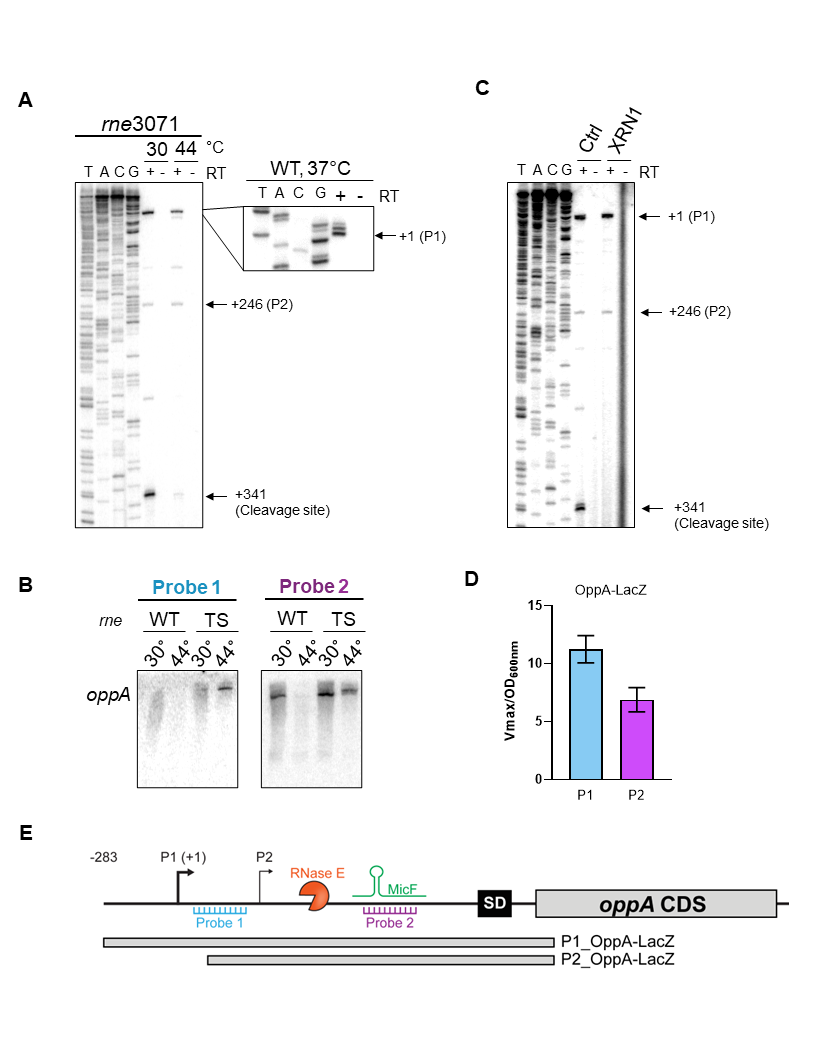


**Figure S3. Analysis of *oppA* transcription start site and 5’UTR processing. (A)** Primer extension assay on RNA samples extracted from a strain expressing the thermosensitive *rne3071* allele, at 30°C or at 44°C. To better map P1, PE was performed on WT strains grown at 37°C and migrated longer. (**B**) Northern blot analysis of *oppA* mRNA. RNA extracted from a strain expressing the thermosensitive *rne3071* allele. Location of probe 1 and probe 2 is indicated on the scheme in **(E)**. For **(A)** and **(B)**, cells were grown at the permissive temperature of 30°C. At OD_600nm_=2.0, half the cells were kept at 30°C and the other half was transferred at the restrictive temperature of 44°C. Incubation carried on for 15 minutes prior to RNA extraction. **(C)** Primer extension assay on RNA samples extracted from WT strains and treated or not with the XRN1 enzyme. **(D)** β-galactosidase assay of OppA-LacZ fusions including P1 and P2 (P1) or just P2 (P2). Samples (N=3, mean ± SD) were taken at OD_600nm_=2.5. **(E)** Schematic representation of *oppA*, including the localisation of the probes used in (C) and the visual representation of the P1 and P2 translational *lacZ* fusions used in (D).


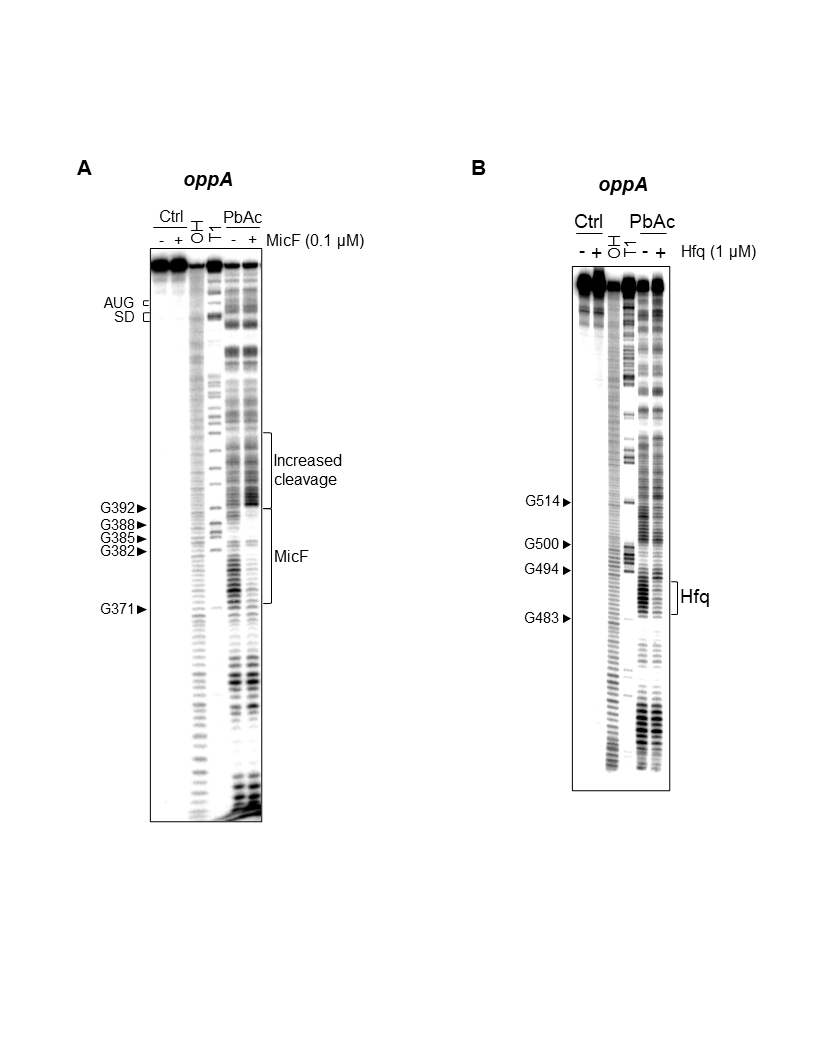


**Figure S4. (A)** Complete lead acetate probing assay gel presented in Figure 2A. Contrast was increased (compared to Figure 2A) to better show the variations in cleavage intensities in presence or absence of MicF. **(B)** Complete footprinting assay gel presented in Figure 5A.


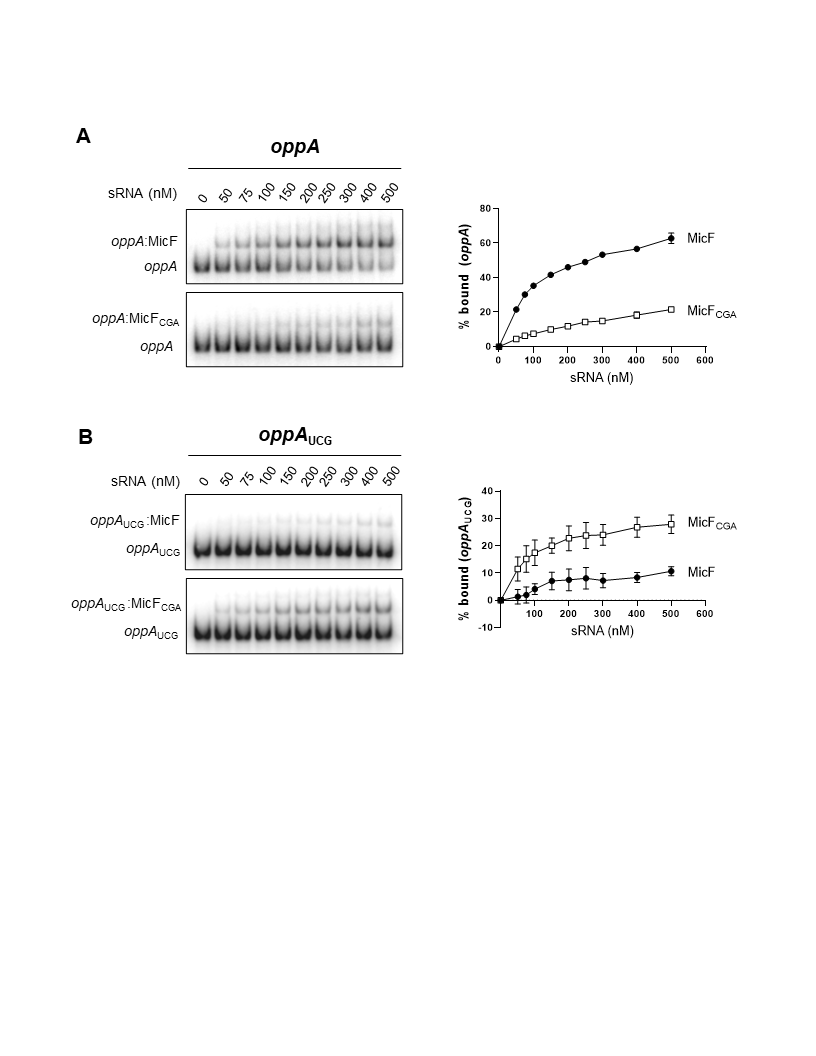


**Figure S5.** Electrophoretic mobility shift assays of **(A)** γ-*oppA* or **(B)** γ-*oppA*_UCG_ incubated in presence of increasing concentrations of MicF or MicF_CGA_ for 15 min. On the right: densitometry analysis (N=2, mean ± SD) of the EMSA plotted as the fraction of γ-*oppA* (top) or γ-*oppA*_UCG_ (bottom) bound to MicF or MicF_CGA_.


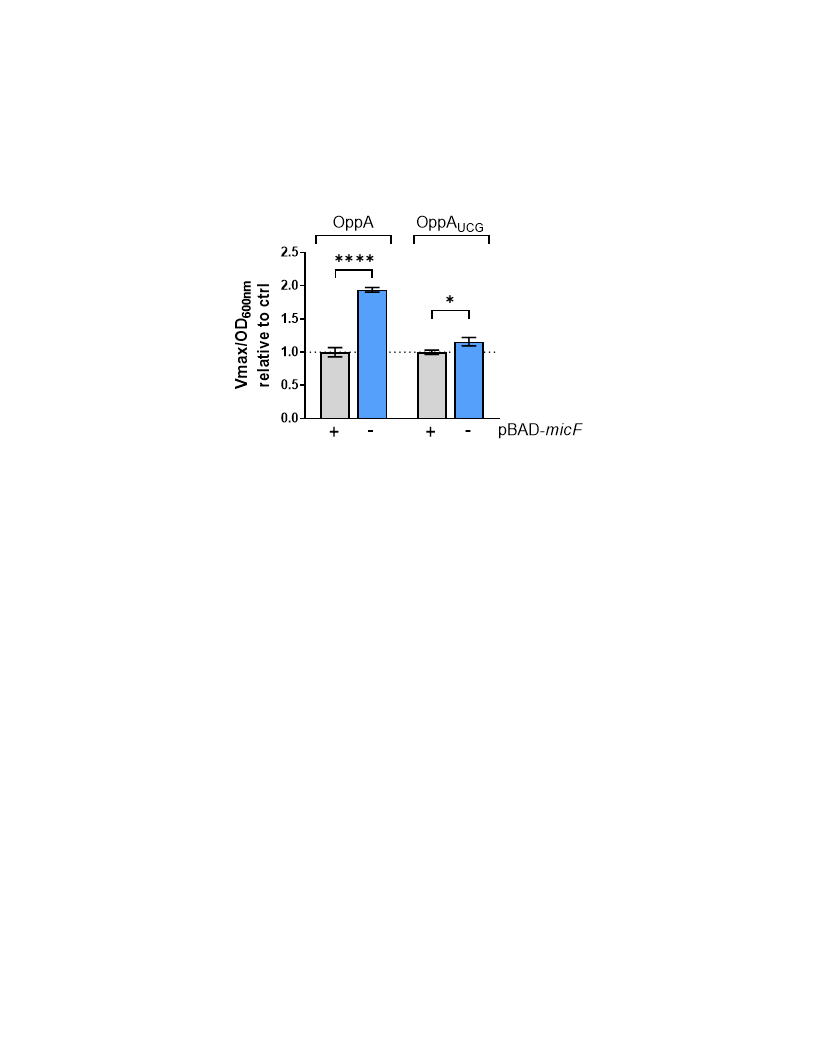


**Figure S6.** β-galactosidase assay of OppA-LacZ (left) or OppA_UCG_-LacZ (right). Expression of *micF* was induced by addition of 0.1% arabinose at OD_600nm_=0.5. Samples (N=3, mean ± SD) were taken at OD_600nm_=2.0. *****p*= 0.00003, ns: *p* = 0.0177, unpaired two-tailed Student’s *t* test.


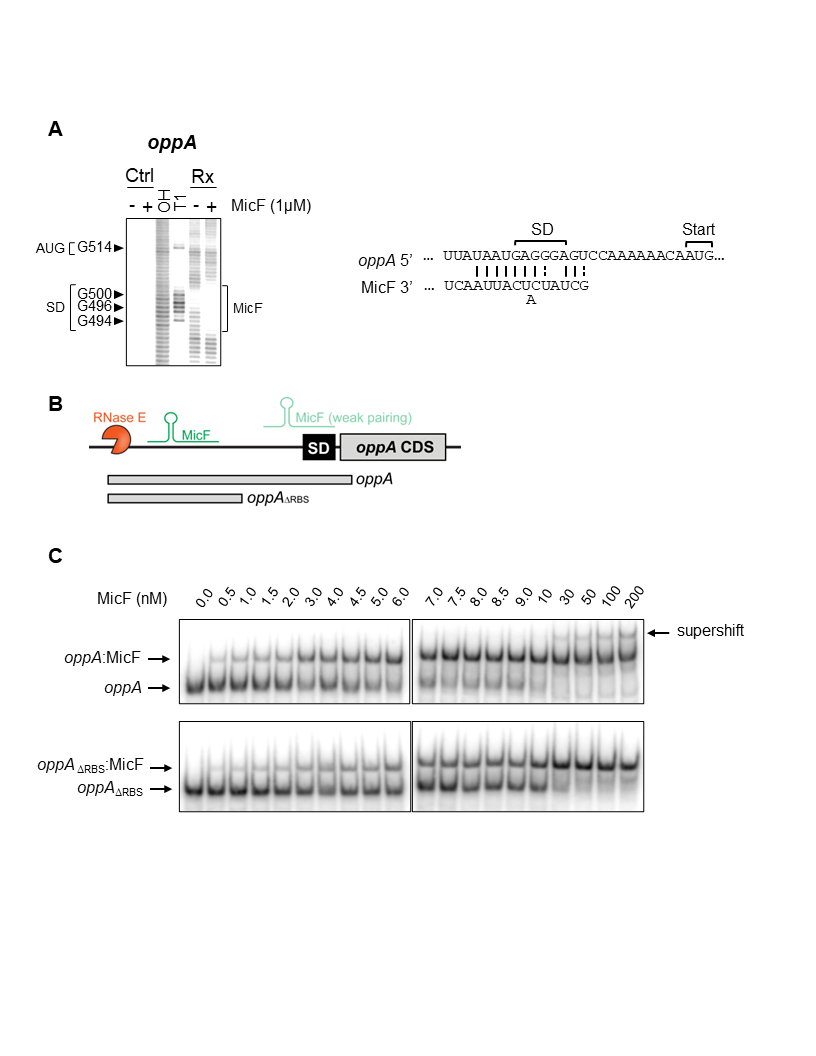


**Figure S7. MicF weakly pairs to the *oppA* Shine-Dalgarno sequence at high concentration in vitro. (A)** Left: in-line probing of γ-*oppA* with MicF. γ-*oppA* was incubated with or without MicF (1 μM) for 48h. Ctrl; non-reacted controls, OH; alkaline ladder, T1; RNase T1 ladder, Rx; In-line experiment. Numbers on the left indicate nucleotide position relative to P1 transcriptional start site. Putative MicF pairing site is indicated with a bracket. The Shine-Dalgarno (SD) is indicated. Right: representation of MicF pairing site near the SD. **(B)** Schematic representation of *oppA* 5’UTR, including the approximate locations of MicF pairing sites. Below, *oppA* and *oppA*_ΔRBS_ used in (C) are represented. **(C)** Electrophoretic mobility shift assays of γ-*oppA* (top) or γ-*oppA*_ΔRBS_ (bottom) incubated in presence of MicF for 15 min, in absence of competitor yeast tRNA. Data is representative of two independent experiments.


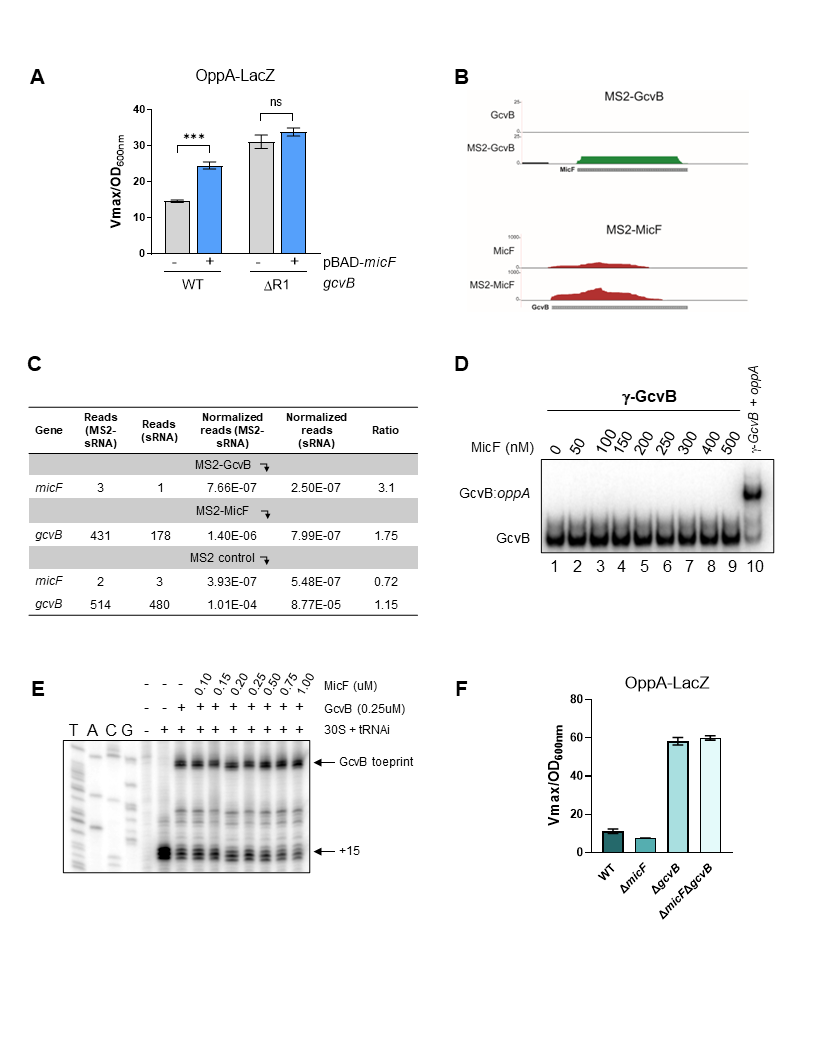


**Figure S8. (A)** β-galactosidase assay of OppA-LacZ in Δ*micF* and Δ*micF/gcvB*ΔR1 backgrounds. Expression of *micF* was induced by addition of 0.1% arabinose at OD_600nm_=0.5. Samples (N=3, mean ± SD) were taken at OD_600nm_=2.0. Data was relativized to the pNM12 empty vector control for each condition. **P*= 0.0454, ****P*< 0.0006, unpaired two-tailed Student’s *t* test. **(B)** Visualization of GcvB and MS2-GcvB MAPS reads for *micF* (top) or of MicF and MS2-MicF MAPS reads for *gcvB* (bottom). **(C)** Information on *gcvB* and *micF* read counts for GcvB, MicF and MS2 MAPS experiments. **(D)** Electrophoretic mobility shift assay of γ-*oppA* incubated in presence of increasing concentrations of MicF for 15 min. Incubation with *oppA* served as a positive control. (E) Toeprinting assay of γ-*oppA* in the presence 0.25µM GcvB and of increasing concentrations of MicF. Lane 1-4; sequencing ladder, lane 5; negative control. Annotation +15 represents the ribosome toeprint. **(F)** β-galactosidase assay of OppA-LacZ in WT, Δ*micF*, Δ*gcvB* and Δ*gcvB*Δ*micF* backgrounds. Samples (N=3, mean ± SD) were taken at OD_600nm_=2.0.


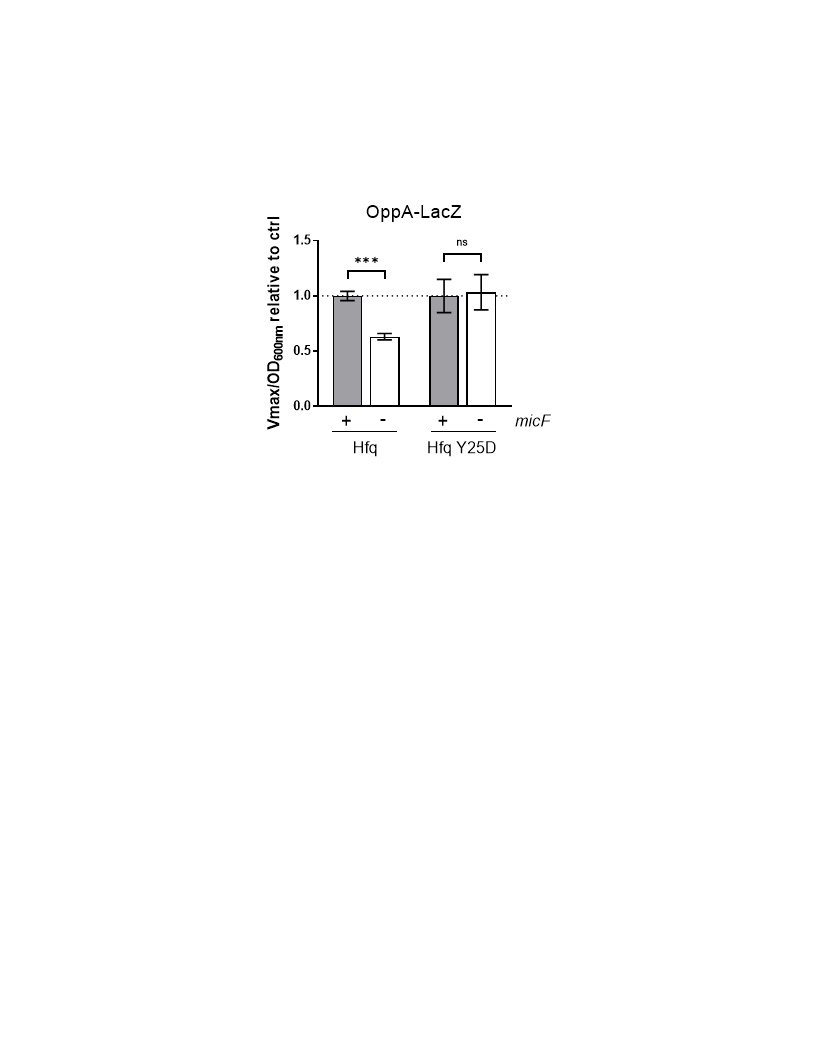


**Figure S9.** β-galactosidase assay of OppA-LacZ in WT and Δ*micF* or in *hfq* Y25D and *hfq* Y25D/Δ*micF* backgrounds. Samples (N=3, mean ± SD) were taken at OD_600nm_=2.0. ****P*= 0.0002, ns: *P* = 0.8027, unpaired two-tailed Student’s *t* test.


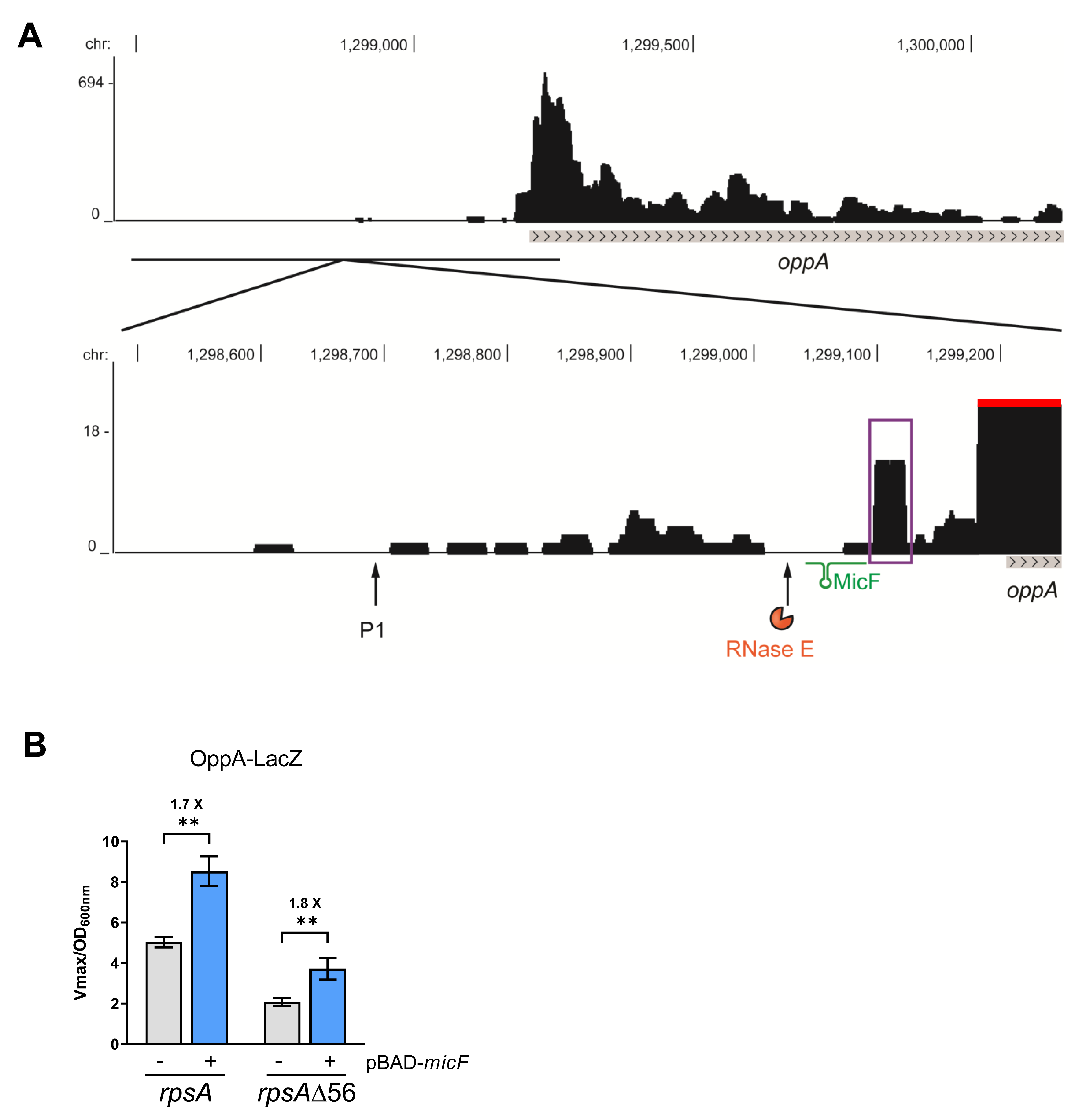


**Figure S10. (A)** Visualization of the reads mapped to *oppA* and *oppA* 5’UTR obtained after a ribosome profiling experiment conducted in WT strains (Geoffroy *et. al.,* in preparation). P1 (+1), the RNase E cleavage side (+341) and MicF pairing site (+372 to +393) are indicated under the graph. The peak corresponding to the putative TE is boxed in purple. For the bottom graph, the reads near the *oppA* start codon (indicated by a red bar) are saturated. **(B)** β-galactosidase assays of OppA-LacZ in Δ*micF* and *rpsA*Δ56/Δ*micF* backgrounds. Expression of *micF* was induced with 0.1% arabinose at OD_600nm_=0.5. Samples (N=3, mean ± SD) were taken 5h after induction. Fold-changes are indicated above. ***P*< 0.01, unpaired two-tailed Student’s *t* test.


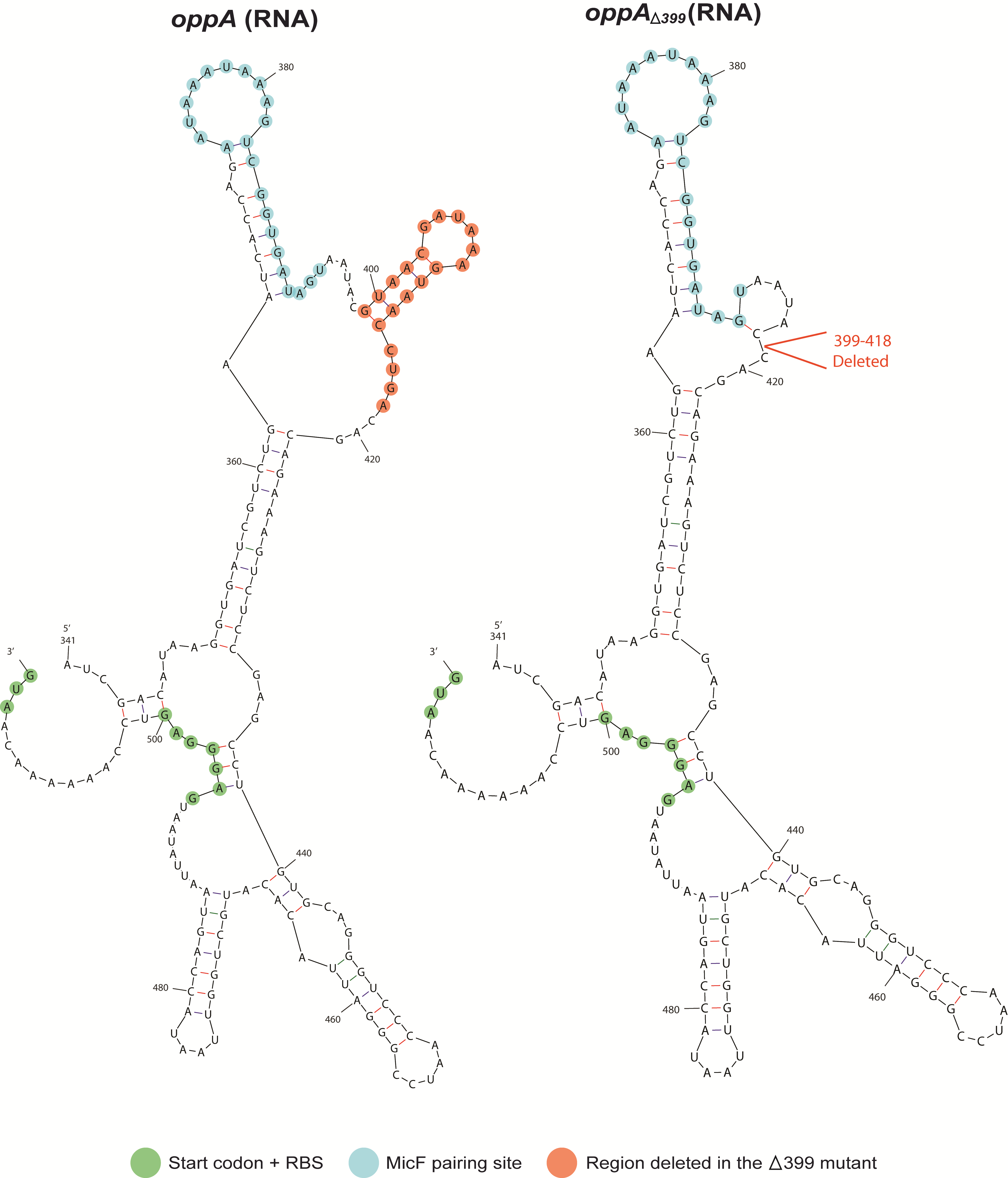


**Figure S11. Secondary structure prediction of WT *oppA* mRNA 5’UTR or *oppA*_Δ399_ mutant 5’UTR**.Structure prediction has been performed using the Mfold web server. In blue; MicF pairing site. In green; RBS and Start codon. In orange; nucleotides deleted in the *oppA*_Δ399_ mutant.
